# An electrochemical advanced oxidation process for the treatment of urban stormwater

**DOI:** 10.1016/j.wroa.2021.100127

**Published:** 2021-11-28

**Authors:** Yanghua Duan, David L. Sedlak

**Affiliations:** Department of Civil & Environmental Engineering, University of California, Berkeley, Berkeley, CA 94720, United States

**Keywords:** Green infrastructure, Aquifer recharge, Electrochemical generation, Hydrogen peroxide, Trace organic contaminant

## Abstract

Recharge of urban stormwater has often been limited by the high cost of land and concerns about contamination of groundwater. To provide a possible solution, we developed an electrochemical advanced oxidation system (UV/H_2_O_2_) that is compatible with high-capacity stormwater recharge systems (e.g., drywells). The system employed an air-diffusion cathode to generate a H_2_O_2_ stock solution (i.e., typically around 600 mM) prior to the storm event. The H_2_O_2_ stock solution was then metered into stormwater and converted into hydroxyl radical (**•**OH) by an ultraviolet lamp. The energy consumption for H_2_O_2_ generation was optimized by adjusting the applied current density and adding an inert salt (e.g., Na_2_SO_4_) to stormwater. H_2_O_2_ in the stock solution was unstable. By mixing the basic H_2_O_2_ containing catholyte and the acidic anolyte, the stability increased, enabling generation of the H_2_O_2_ stock solution up to three days prior the storm event with loss of less than 20% of the H_2_O_2_. Lab-scale experiments and a kinetic model were used to assess the feasibility of the full-scale advanced oxidation system. System performance decreased at elevated concentrations of dissolved organic carbon in stormwater, due to enhanced light reflection and backscattering at the water-air interface in the UV reactor, competition for UV light absorption with H_2_O_2_ and the tendency of organic matter to act as a **•**OH scavenger. The proposed system can be incorporated into drywells to remove greater than 90% of trace organic contaminants under typical operating conditions. The electrical energy per order of the system is estimated to range from 0.5 to 2 kWh/m^3^, depending on the dissolved organic carbon concentration.

## Highlights

• Pre-production of concentrated H_2_O_2_ before treatment reduced the system footprint.
• System design was optimized based on H_2_O_2_ generation, storage, and activation.
• Optimization reduced cost and footprint of electrochemical stormwater treatment.

## Graphical abstract

**Figure:**
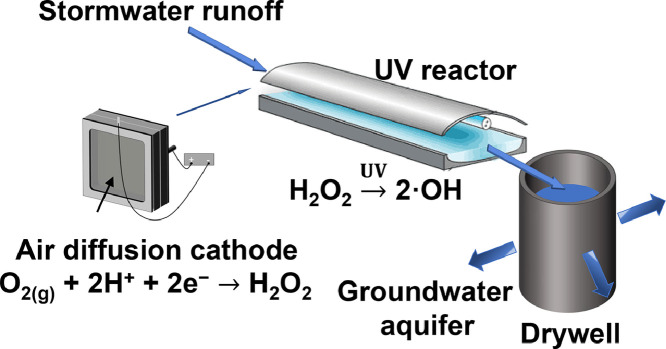

## Introduction

Climate change and rapid population growth are exacerbating water scarcity in cities around the world (Flörke et al., 2018). The capture and use of urban stormwater are attractive to water-stressed cities because this underutilized water source is available in large quantities and does not require large investments in environmentally damaging, long-distance conveyance systems. Due to limited opportunities for water storage within cities, captured urban runoff might best be used for aquifer recharge in cities that rely upon groundwater (Luthy et al., 2019). However, stormwater contains contaminants including pesticides, compounds released by vehicles and waterborne pathogens, all of which can pose risks to drinking water supplies (Grebel et al., 2013; Rippy et al., 2017; Spahr et al., 2020). Rather than relying upon natural attenuation or treatment after extraction to remove trace organic contaminants, some form of treatment may be appropriate prior to recharge (Zhang et al., 2020).

Researchers have studied the use of engineered geomedia, such as manganese oxide-coated sands (Charbonnet et al., 2020; Grebel et al., 2016), woodchips (Ashoori et al., 2019), functionalized clays (Ray et al., 2019) and biochar (Ashoori et al., 2019; Boehm et al., 2020; Ulrich et al., 2015) as a passive means of stormwater treatment. However, such treatment systems are often limited by the low hydraulic conductivities of geomedia or underlying soils and sediments (Barnes et al., 2014; Minnesota Stormwater Steering Committee 2005; Ray et al., 2019). Infiltration rates are also limited by the need to achieve adequate contact times between the engineered geomedia and the contaminants (Grebel et al., 2016; Shimabuku et al., 2016). In addition, geomedia eventually will clog or be exhausted, which necessitates replacement or regeneration to assure continued operation (Charbonnet et al., 2018, 2021). As a result, infiltration-based techniques that employ geomedia are often unable to treat large volumes of stormwater in urban areas.

In locations where surface infiltration is impractical, drywells offer an attractive alternative due to their high infiltration rates and small footprints (Edwards et al., 2016). For reference, to achieve a recharge rate of 400 L/min, a value typical of drywells, an area of 1.5 m^2^ and 1200 m^2^ would be required for drywells and rain gardens (maximum infiltration rate = 2 cm h^−^^1^), respectively (Hunt et al., 2010; Prince George's County, Maryland 1999, Torrent Resources, 2021). However, it has been difficult to incorporate treatment systems capable of removing trace organic compounds and viruses into drywells because high flow rates during storms often preclude the use of geomedia. As a result, the use of drywells for urban stormwater infiltration without any form of treatment has contaminated groundwater with volatile organic compounds, benzene, and petroleum hydrocarbons (City of Portland Bureau of Environmental Services 2008; Jurgens et al., 2008; Lindemann 1999).

Advanced oxidation processes (AOPs) such as UV/H_2_O_2_ have been used for over 40 years to oxidize organic contaminants in hazardous waste, drinking water and contaminated groundwater by taking advantage of the high reactivity and low selectivity of hydroxyl radical (**•**OH) (Koubek 1977). Furthermore, the process also controls waterborne pathogens because the fluence of ultraviolet (UV) light used in the process is typically about ten times higher than values used to disinfect drinking water (Sun et al., 2016). Despite its potential applicability, only a few attempts (Bettman 2020; Zheng et al., 2021) have been made to employ AOPs in stormwater treatment systems due to the cost and challenges associated with system maintenance and reagent replenishment, especially when infiltration structures are distributed around a city.

Electrochemical advanced oxidation processes (EAOPs) are promising options for decentralized system because of their modular design, high efficiency, and ease of automation (Feng et al., 2018; Kanakaraju et al., 2018; Miklos et al., 2018; Moreira et al., 2017; Nidheesh et al., 2018; Yang 2020; Yang and Hoffmann 2016). To overcome the difficulties associated with using AOPs for distributed water treatment, we employed an inexpensive, compact system that generates H_2_O_2_ by electrochemical reduction of O_2_ (eq (1)) with an air-diffusion cathode that uses only electricity and passive diffusion of air.

(1) $$O_{2}+2e^{-}+2H^{+}\to H_{2}O_{2}{\;E}_{H}^{{}^{\circ }\;}=+0.70V\;$$

The H_2_O_2_ is then converted to **•**OH by exposure to UV light (eq (2)) in a low-cost reactor that was originally developed for drinking water disinfection in resource limited communities (Gadgil 2008).

(2) $${\text{H}}_{2}O_{2}\overset{\;\text{UV}\;}{\to }2•\mathrm{OH}$$

To employ this approach in a drywell or another type of stormwater infiltration system, a sufficient quantity of H_2_O_2_ must be generated using inexpensive equipment that can fit into a small area. To accomplish this objective, we developeddeveloped H_2_O_2_ generation and storage strategies to produce a H_2_O_2_ stock solution prior to the storm event (potentially with stormwater captured previously) and then metered the H_2_O_2_ stock solution into stormwater prior to exposing it to UV light. Using a kinetic model of H_2_O_2_ activation and contaminant transformation, we assessed the feasibility of employing UV/H_2_O_2_ to treat urban stormwater under conditions typically encountered in drywells. Finally, by systematic optimization of each process, we demonstrated EAOP is feasible for distributed stormwater treatment.

## Materials and methods

### Materials

All experiments were performed at room temperature (23 ± 2°C) with chemicals of reagent grade or higher (Sigma-Aldrich, St. Louis, MO). Suwannee River natural organic matter (reverse osmosis isolate) was obtained from the International Humic Substances Society (St. Paul, MN). Ultrapure water from a Milli-Q system (R > 18 MΩ) was used for all experiments except when specifically noted.

Simulated stormwater was made in the manner described by Grebel et al. (2016). The composition of the water (Table S1) was chosen to represent the main solutes in urban runoff. In a previous study, this matrix exhibited similar performance to that observed in authentic stormwater (Grebel et al., 2016). In advanced oxidation process experiments (described later in the Materials and methods Section), a synthetic humic acid sodium salt (Sigma-Aldrich, St. Louis, MO) and deionized water (Culligan, Rosemont, IL) were used rather than Suwanee River natural organic matter and Milli-Q water because of the high cost of preparing the large volumes of water to run the experiments (i.e., about 9 L of synthetic stormwater was needed to assess each configuration of the treatment system). All solutions containing organic carbon (i.e., humic acid or natural organic matter) were prepared from a concentrated organic carbon stock solution (typically around 100 mg of chemicals/L), which was filtered through 0.45-μm glass fiber filters (Tisch Scientific, Cleves, OH) prior to use.

### Electrochemical cell and UV reactor

Electrolysis experiments were performed in a two-chambered parallel plate electrochemical cell modified from the device described by Barazesh et al. (2015). Briefly, the electrochemical cell was made of acrylic plastic (McMaster-Carr, Los Angeles, CA) and equipped with a Pt-coated Ti mesh anode (dimensions: 5.1 × 7.6 cm; TWL, USA) and a homemade air-diffusion cathode (dimensions: 4.0 × 4.0 cm) as described by Barazesh et al. (2015). Graphite powder (200 mesh, Alfa Aesar, Ward Hill, MA) and PTFE were coated on the air-facing side of the cathode. Carbon black (Cabot Black Pearls 2000, Cabot, Boston, MA) and PFTE were coated on the liquid-facing side with propanol as solvent. The cathodic chamber and anodic chamber were separated by a cation exchange membrane (Ultrex CMI-7000, Membranes International Inc., Ringwood, NJ). The thickness of each chamber was 1.7 cm. The divided electrochemical cell was chosen to avoid anodic oxidation of H_2_O_2_, which could limit the generation of high concentration H_2_O_2_ solutions and lower the Faraday efficiency of the generation process (Abdullah and Xing 2017; Ma et al., 2019; Panizza and Cerisola 2008; Pérez et al., 2017).

After H_2_O_2_ was generated in the electrochemical cell, the H_2_O_2_-containing catholyte was mixed with the anolyte to form a H_2_O_2_ stock solution. The H_2_O_2_ stock solution was then diluted into simulated stormwater and passed through a UV reactor. The UV reactor used in this study was described by Gadgil and Garud (1998). Briefly, the reactor had a volume of 5.5 L and included a 60-watt low-pressure UV lamp (Philips, Andover, MA) mounted under an aluminum reflector. The UV lamp was situated approximately 6 cm above the surface of the flowing water.

### H_2_O_2_ generation and energy consumption

Batch experiments to assess H_2_O_2_ generation were conducted in Na_2_SO_4_ electrolyte or in simulated stormwater amended with Na_2_SO_4_. Equal volumes of solution were circulated in the two-cell chambers at a flow rate of 30 mL/min with a peristaltic pump (Masterflex, Vernon Hills, IL). Electrolysis experiments were performed at fixed currents controlled by a direct current power supply (B&K Precision, Yorba Linda, CA). The air-diffusion cathode was tested under different current densities (200 to 1200 A/m^2^) and replaced if any leaks were detected or a low initial Faraday efficiency (i.e., below around 70%) was measured. Despite the decreased performance of the electrode over time, the electrode performance was relatively stable for several runs of operation (at least tens of hours). All H_2_O_2_ generation experiments were conducted at least in triplicate (*n* = 3–6).

The applied charge density ($\rho_{q},\;C/L)$ was calculated based on the current density ($i,\;A/m^{2})$, electrode area $\left(A,m^{2}\right)$, electrolysis time (t,s), and catholyte volume (V_ca_, L):

(3) $$\rho_{q}=\underset{0}{\int^{t}}\frac{i\;\times \;A}{V_{\mathrm{ca}}}\text{dt}=\frac{\text{iAt}}{V_{\text{ca}}}\;$$

The energy consumed during H_2_O_2_ generation was calculated based on the measured H_2_O_2_ concentration ([H_2_O_2_], M), catholyte volume (V_ca_, L), electrode area (A,m^2^), electrolysis time (t,s), current density ($i,\;A/m^{2})$, and cell voltage (V_cell_, V):

(4) $$\text{Energy}\;\left(\frac{\text{kWh}}{\text{mg}\;H_{2}O_{2}}\right)=\;\frac{\int_{0}^{t}iV_{\text{cell}}\text{Adt}}{\left[H_{2}O_{2}\right]V_{\text{ca}}}\frac{1\text{mo}l\;H_{2}O_{2}}{34,000\text{mg}\;H_{2}O_{2}}\frac{1\;\text{kWh}}{3.6\;\times 10^{6}\;J}\;$$

### H_2_O_2_ stability

The stability of the H_2_O_2_ stock solution was assessed before and after mixing the catholyte with the anolyte. The H_2_O_2_ was generated under 800 A/m^2^ of applied current density in batch mode with Na_2_SO_4_ electrolyte or Na_2_SO_4_-amended simulated stormwater. For the mixed electrolyte storage, catholyte and anolyte were mixed slowly with a peristaltic pump. To examine the effect of dissolved solids on H_2_O_2_ stability, 50 mg/L of San Joaquin soil (NIST SRM 2709a, Gaithersburg, MD) was added to the mixed simulated stormwater (Mean TSS in urban stormwater = 58 mg/L as described by Grebel et al., 2013) prepared as described in Text S1.

### Advanced oxidation process

Experiments to assess the performance of the AOP were conducted in a simulated stormwater solution amended with different concentrations of Sigma humic acid (0–5 mg-C/L). During each experiment, 9 L of simulated stormwater amended with 10 μg/L of carbamazepine (CBZ) was circulated between a HDPE bucket (M&M Industries, Chattanooga, TN) and the UV reactor (*V* = 5.5 L) with a submersible pump operated at a flow rate of 7.6 L/min (Figure S1). By circulating the stormwater, we were able to simulate the performance of an upscaled UV reactor consisting of multiple UV reactors in series without the complexity of operating the large system under laboratory conditions. The H_2_O_2_ stock solution was diluted from a 0.18 ± 0.03 M (*n* = 15) stock solution by mixing equal volumes of catholyte and anolyte generated by batch-mode electrolysis of a 0.2 M Na_2_SO_4_ electrolyte solution. Although CBZ has not been detected frequently in stormwater, it was chosen as a surrogate for trace organic contaminants in stormwater because it is mainly transformed by reactions with **•**OH radicals; it exhibits a low rate of direct photolysis and does not react with other photo-produced transients produced by UV light (Barazesh et al., 2015). Most stormwater contaminants (e.g., pesticides) exhibit similar rate constants to CBZ for reactions with •OH radical. Some compounds also may be transformed by direct photolysis and/or transient species produced when natural organic matter is exposed to UV light (Table S2). For some compounds that react with •OH radicals slower than CBZ (e.g., atrazine), the overall removal rates were observed to be higher than that of CBZ during the UV/H_2_O_2_ process because the other compounds undergo direct photolysis (Rozas et al., 2016). Therefore, CBZ can be used as a surrogate for most of the trace organic contaminants commonly detected in stormwater.

The light field in the UV reactor was characterized by chemical actinometry using atrazine under the same experimental conditions. The solution was amended with 100 μg/L of atrazine (Ɛ_254_ = 3860 M^−^^1^ cm^−1^, Φ_254_ = 0.046 mol Ei^−1^); the same concentrations of humic acid was used (0–5 mg-C/L). The solution was buffered at pH = 7 using a 5 mM phosphate buffer (Bolton and Linden, 2003, Canonica, Meunier and Von Gunten, 2008). The light fluence, which was measured before and after the advanced oxidation experiments, varied by less than 5%. Details of the calculation of light fluence are included in the Supplementary Information Section.

### Analytical methods

H_2_O_2_ was measured with a titanium (IV) sulfate method modified from Eisenberg (1943) with a Shimadzu UV-2600 spectrophotometer at 420 nm. H_2_O_2_ was measured within 5 min of sampling to minimize artifacts caused by H_2_O_2_ decomposition. Total organic carbon (TOC) was measured using a Shimadzu TOC-V analyzer. CBZ-containing samples were filtered through 0.22-μm glass fiber filters prior to adding isotopically labeled internal standard and 100 μL of methanol to quench any possible **•**OH reactions that could consume CBZ. CBZ was quantified in multiple reaction monitoring (MRM) mode with an Agilent 1200 series HPLC system coupled to a 6460 triple quadrupole tandem mass spectrometer (HPLC-MS/MS) within 24 h after sampling (Jasper et al., 2014). Metal ions were quantified in triplicate on an Agilent 7700 Series Inductively Coupled Plasma-Mass Spectrometer (ICP-MS).

### Kinetic model for the UV/H_2_O_2_ treatment

A kinetic model was employed based on reaction schemes developed in previously published studies (Crittenden et al., 1999; Glaze et al., 1995; Song et al., 2008) using Kintecus version 6.80 (Ianni 2003). Details of the model construction are described in the Supplementary Information Section.

## Results and discussion

### H_2_O_2_ generation as a function of current density

The applied current density could affect the H_2_O_2_ generation performance. For stormwater treatment, an ideal electrode should be able to generate a high concentration of H_2_O_2_ stock solution with high Faraday efficiency. Comparing to in situ generation of H_2_O_2_ in a flow-through reactor (Barazesh et al., 2015), generating a high concentration of H_2_O_2_ before a storm event and storing it could reduce the energy consumption and system footprint by reducing the applied current density and electrode area. It could also minimize the amount of salt added to the treated stormwater (when H_2_O_2_ is produced in a salt-amended electrolyte solution as described below). However, at very high concentrations, especially under basic pH conditions encountered in the catholyte (pH around 11.5, Figure S4), the H_2_O_2_ is unstable due to self-decomposition and reactions with transition metals and organics (Galbács and Csányi 1983; Petigara et al., 2002). Under applied current, H_2_O_2_ can also undergo electrochemical reduction to H_2_O (Jiang et al., 2018). Therefore, development of a better understanding of the effect of current density on H_2_O_2_ production rate, Faraday efficiency and factors affecting the maximum H_2_O_2_ concentration that can be generated provides a basis for selecting an optimum operational mode.

**Fig. 1:**
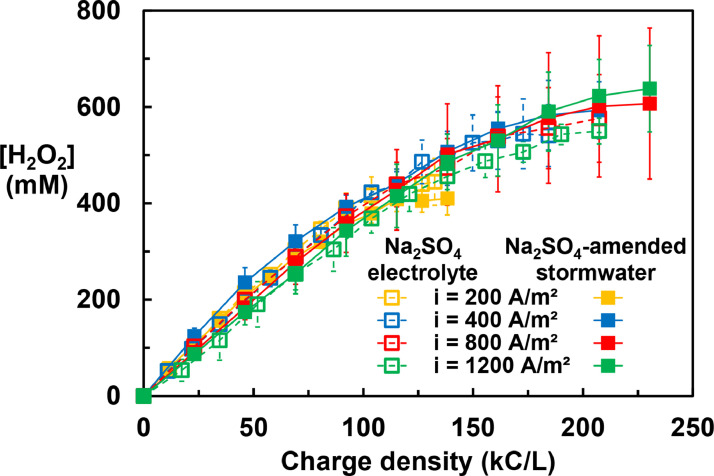
Production of H_2_O_2_ during electrolysis in 0.2 M Na_2_SO_4_-amended stormwater and 0.2 M Na_2_SO_4_ electrolyte at varying current densities. Error bars represent one standard deviation.

Hydrogen peroxide formation in the catholyte (Fig. 1) exhibited similar behavior in Na_2_SO_4_ electrolyte and in Na_2_SO_4_-amended stormwater at current densities ranging from 200 to 1200 A/m^2^. The H_2_O_2_ concentrations increased in approximately a linear fashion with the applied charge density up to about 120 kC/L, at which point a concentration of around 430 mM had been produced. For electrolysis conducted with 200 A/m^2^ of current density, the H_2_O_2_ concentration remained at around 430 mM as the charge density further increased. For electrolysis conducted with higher current densities, after the charge density reaching 120 kC/L, the H_2_O_2_ generation efficiency decreased until the H_2_O_2_ concentration in catholyte plateaued at around 600 mM. Because of the similar performance with respect to applied current density, the system design and operation adjustment can be significantly simplified, leading to considerable flexibility in the operation of the treatment system.

To assess the performance of this air-diffusion cathode for H_2_O_2_ generation at high H_2_O_2_ concentrations, these data were compared with results from previous studies on electrochemical synthesis of H_2_O_2_ in terms of surface normalized production rate, Faraday efficiency, and maximum H_2_O_2_ concentration (Table S7). The device tested in this study was better suited for producing a concentrated stock solution for use in water treatment than the other 12 systems reported in the literature that also can generate a relatively high concentration of H_2_O_2_ solution with relative high Faraday efficiency. This may be due to the fact that most previous efforts either focused on achieving the maximum possible H_2_O_2_ concentration ([H_2_O_2_]_max_ ranged from 320 to 5500 mM; Faraday efficiency ranged from 12 to 60%) (Iwasaki et al., 2018; Luo et al., 2015; Oloman and Watkinson 1979; Yamanaka et al., 2008; Yamanaka and Murayama 2008; Yamanaka et al., 2006) or high Faraday efficiency (Faraday efficiency ranged from 79 to 99%; [H_2_O_2_]_max_ ranged from 24 to 250 mM) (Chen et al., 2017; Li et al., 2013; Sa et al., 2019; Wang et al., 2020). Li et al. (2020) achieved 980 mM of [H_2_O_2_]_max_ with a Faraday efficiency of 79%. But their fuel cell reactor inhibits the application of their device in distributed locations because of lack of supply of pure H_2_ and O_2_ under the actual field condition. In addition, most of those devices were designed to be operated in strongly acidic or alkaline conditions, with deteriorating performances observed at lower acid/base concentrations (Foller et al., 1991; Yamanaka et al., 2008). Among the other approaches, the method of Xia et al. (2019) shows considerable promise due to its high Faraday efficiency (90 to 95%) and ability to produce a high concentration of H_2_O_2_ (5900 mM). However, the system requires pumping humidified air as a source of O_2_ rather than relying on the passive diffusion of air. The system also requires passage of water through a solid electrolyte. These requirements would likely increase the system cost and increase the risk of system failures, two outcomes that could be problematic in remote operations.

### Energy consumption during H_2_O_2_ generation

The energy consumption was dictated by the cell voltage, which was mainly affected by the ohmic loss and the overpotential. The ohmic loss of an electrochemical system depends upon the conductivity of the solution; the overpotential is the driving force of the electrochemical process and was mainly determined by the electrode material and the applied current density.

For low-conductivity solutions (e.g., stormwater), the energy consumption was mainly affected by the low conductivity of the electrolyte. Therefore, it is possible to reduce the energy consumed for H_2_O_2_ production by adding an inert electrolyte (e.g., Na_2_SO_4_) to the stormwater. Na_2_SO_4_ was chosen as the electrolyte amendment because of its relatively low cost (about $100/ton) and the absence of undesirable products (e.g., ClO_3_^–^ from NaCl oxidation) produced when it passes through the anode chamber. A Na_2_SO_4_ concentration of 0.2 M was chosen to strike a balance between energy consumption and the amount of salt added to the stock solution (energy consumption in stormwater amended with 0.02 M to 0.2 M of Na_2_SO_4_ was estimated as described in the Text S4 and Figure S6). At lower salt concentrations ([Na_2_SO_4_] < 0.1 M), the energy consumption increased dramatically as the salt concentration decreased. At [Na_2_SO_4_] > 0.1 M, the addition of salts barely decreased the energy consumption because the overpotential dominated the cell voltage. Given the relatively low cost of the Na_2_SO_4_ comparing to the cost of electricity, the estimated overall cost of the system was decreased by adding Na_2_SO_4_ into stormwater used for production of the H_2_O_2_ stock solution (Text S5 and Figure S8). For realistic operating conditions, the amount of salts can be determined by balancing the energy cost and the cost for delivering and dosing the salts into the solution.

Adding an electrolyte to a stock solution prior to H_2_O_2_ generation and then mixing a small volume of concentrated H_2_O_2_ solution into the stormwater would increase the total dissolved solids (TDS) of the treated stormwater. To avoid adverse impacts on water quality, it is important to assure that the TDS and sulfate concentration of the treated water would not exceed the USEPA secondary maximum contaminant levels (SMCLs) for TDS of 500 mg/L as well as the sulfate concentration limit of 250 mg/L (USEPA 2017). If the target H_2_O_2_ concentration in the stock solution was 200 mM and a 0.2 M solution of Na_2_SO_4_ was used in the stock solution, then addition of H_2_O_2_ to produce an initial H_2_O_2_ concentration of 1 mM in the stormwater will increase the TDS by about 140 mg/L and sulfate by around 100 mg/L which would be acceptable for drinking water purposes, provided that the initial stormwater TDS and sulfate concentrations were below 360 mg/L and 150 mg/L, respectively. If this were not the case, a lower concentration of Na_2_SO_4_ would be necessary.

**Fig. 2:**
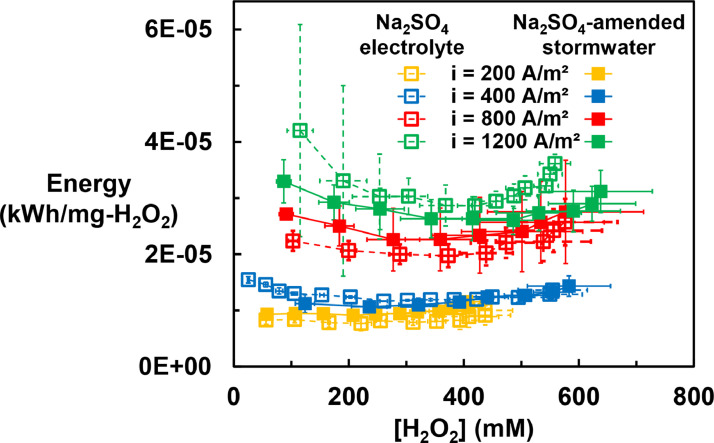
Energy consumption of H_2_O_2_ generation under different applied current densities for 0.2 M Na_2_SO_4_-amended stormwater and 0.2 M Na_2_SO_4_ electrolyte. Error bars not shown are smaller than symbols.

The energy consumption increased approximately in a linear fashion with respect to the applied current density (Fig. 2 and Figure S9). The increasing in energy consumption with increased current density was due to the increased ohmic loss and overpotential. The solution matrix (i.e., the presence or absence of synthetic stormwater constituents) had little effect on the energy consumption because of the similar solution conductivity and H_2_O_2_ generation performance between Na_2_SO_4_-amended simulated stormwater and Na_2_SO_4_ electrolyte. For electrolysis performed with current density of 800 A/m^2^ and 1200 A/m^2^, the energy consumption increased slightly after the H_2_O_2_ concentration reaching 450 mM due to decreased H_2_O_2_ generation efficiency.

Although high current density may reduce the lifetime of the electrode (Carlesi Jara and Fino 2010), it enables H_2_O_2_ generation with a smaller electrode, which could lower the system capital cost. Use of high current densities could also generate H_2_O_2_ more quickly, meaning that less time would be needed to generate a sufficient quantity of H_2_O_2_ to prepare for a storm. Operating at a low current density could lower the energy consumption by over 50% but H_2_O_2_ loss during storage might become more significant under such conditions (see following section). The current density employed in the system can be adjusted based on uncertainty in the weather forecast, the predicted duration and intensity of the storm and the amount of time that has elapsed since the previous storm, which often affects stormwater quality (Grebel et al., 2013).

Due to the voltage limitation of the power supply used in this research, the energy consumption was evaluated with Na_2_SO_4_ concentrations above 0.02 M. Given the ionic strength of the simulated stormwater is typically around 0.005 M, use of the air-diffusion cathode to produce a stock solution of H_2_O_2_ without addition of salts would require a lower applied current density. Further research on electrode performance on H_2_O_2_ generation under low current density conditions is needed to evaluate the tradeoffs of operating this system without added salts.

### H_2_O_2_ stability during storage

After production, H_2_O_2_ may need to be stored for periods of several days. The stability of H_2_O_2_ maybe affected by solution pH (Galbács and Csányi 1983; Watts et al., 1999), transition metals (Haber and Weiss 1934; McKee 1969), organic matter (Petigara et al., 2002; Romero et al., 2009), soil particles (Petigara et al., 2002), and enzymes (Netto et al., 1996). At the high concentration of H_2_O_2_ employed in this system, microbial activity should be relatively low (Collén and Pedersén 1996). Therefore, the loss caused by peroxidase enzymes should be insignificant in this system.

**Fig. 3:**
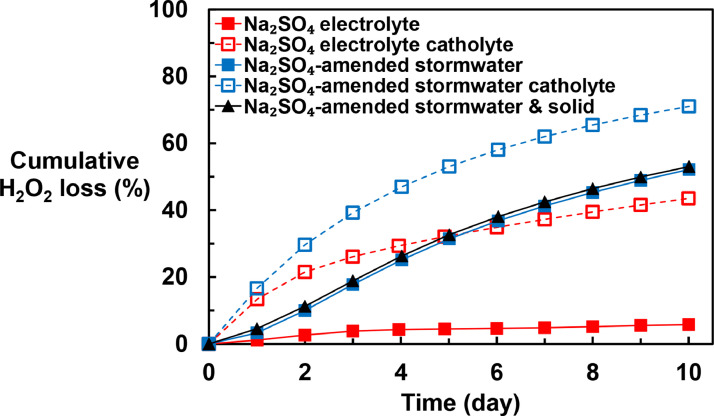
Predicted cumulative loss of H_2_O_2_ during storage of stock solutions prepared in advance of a storm event.

Previous research has indicated that H_2_O_2_ is unstable under alkaline pH conditions (Galbács and Csányi 1983; Nicoll and Smith 1955; Špalek et al., 1982). Therefore, the stability of H_2_O_2_ solution was assessed in the basic catholyte (initial pH around 12) and after the catholyte was mixed with acidic anolyte (which had an initial pH = 0.61 ± 0.04 and 0.78 ± 0.15 for Na_2_SO_4_ electrolyte and simulated stormwater, respectively) generated during the electrolysis process (referred to as “Na_2_SO_4_ electrolyte” and “Na_2_SO_4_-amended stormwater” in Figure S10, Figure S11 and Fig. 3). The concentration of H_2_O_2_ decreased by 33% in Na_2_SO_4_-amended stormwater catholyte and 27% in Na_2_SO_4_ catholyte within a day. When it was mixed with the anolyte, only 7% and 2% of the H_2_O_2_ was lost within a day in the mixed Na_2_SO_4_-amended stormwater and the mixed Na_2_SO_4_ electrolyte, respectively (Figure S10). The enhanced stability was attributed to the reduction in the solution pH (Figure S11). Abel (1952) and Duke and Haas (1961) reported the homogeneous, uncatalyzed decomposition rate and reaction mechanism of H_2_O_2_ in alkaline aqueous solution as described in eq (5)-(6):

(5) $$H_{2}O_{2}⇌{\text{HO}}_{2}^{-}\;+\;H^{+}\;\;K=\;10^{-11.7}$$

(6) $$H_{2}O_{2}\;+\;{\text{HO}}_{2}^{-}\to {\mathrm{OH}}^{-}\;+\;O_{2}\;+\;H_{2}O\;K=1.61\;{\text{M}}^{-1}{\text{h}}^{-1}$$

During the H_2_O_2_ generation, the high concentration of H_2_O_2_/HO_2_^–^ in the catholyte buffered the solution pH around the pK_a_ value of H_2_O_2_ (eq (5)). Without further adjusting the pH, the disproportionation reaction (eq (6)) could be an important loss mechanism.

Although neutralization of the catholyte slowed the rate of H_2_O_2_ decomposition, rates of H_2_O_2_ decomposition in the catholyte/anolyte mixture increased substantially after about one day. The most likely explanation for the loss of H_2_O_2_ was transition metal-catalyzed processes, particularly from Fe and Cu in the Suwannee River natural organic matter (Table S8). The reaction mechanisms for H_2_O_2_ decomposition in simulated stormwater matrix is complicated because of the interactions of H_2_O_2_ with transition metals (Haber and Weiss 1934; McKee 1969), organic matter (Petigara et al., 2002; Romero et al., 2009; Walling and Goosen 1973) and phosphate (Kakarla and Watts 1997) and is therefore beyond the scope of this study. The measured concentrations of Fe and Cu in Na_2_SO_4_-amended simulated stormwater were similar to the concentrations observed in stormwater samples collected when suspended particle concentrations were low, as would be expected after stormwater-pretreatment ahead of drywell infiltration (Kabir et al., 2014; Li et al., 2012). Most of the Fe and Cu in stormwater is likely to be associated with natural organic matters or complexed by phosphate (Aiken et al. 2011, Benjamin 2014, Rose and Waite 2003). Therefore, the H_2_O_2_ stability observed in the Na_2_SO_4_-amended simulated stormwater should represent its stability under actual field conditions. Addition of 50 mg/L of San Joaquin soil, which is a typical concentration of suspended solids concentrations in stormwater (Mean TSS = 58 mg/L; Grebel et al., 2013), did not affect H_2_O_2_ stability.

To minimize H_2_O_2_ loss during storage, the H_2_O_2_ generation process ideally should be completed immediately before the storm event starts. Assuming the H_2_O_2_ is generated in a continuous flow-through electrochemical module that has a hydraulic residence time of less than 1 hour, most of the loss of H_2_O_2_ will occur in the storage tank. The loss will be driven by the H_2_O_2_ generated at the beginning of the generating process which will be stored for longer time. The cumulative H_2_O_2_ loss under different operating conditions (Fig. 3) was calculated based on the results of decomposition experiments (Figure S10) and the storage time (T, day):

(7) $$\mathrm{Cumulative}\;H_{2}O_{2}\mathrm{loss}\left(\%\right)=\frac{\int_{0}^{T}\left(1-\frac{C}{C_{0}}\right)\text{dt}}{T}\times 100\%$$

To limit the overall loss of H_2_O_2_ to less than 20%, several approaches are possible. First, H_2_O_2_ generated by passage of water through the cathode can be used immediately, if H_2_O_2_ generation starts less than one day prior to the storm event. Alternatively, stormwater from the cathode chamber can be neutralized by mixing with stormwater that has passed through the anode, in which case the H_2_O_2_ generation can be started up to three days prior to the storm event. Finally, water with fewer impurities (e.g., tap water), can be used for H_2_O_2_ generation, in which case, generation of H_2_O_2_ can take place up to 10 days prior to the storm event.

An effective H_2_O_2_ generation strategy can be formulated based on the performance of the air-diffusion cathode and the stability of H_2_O_2_. The required total current was estimated from the stability of H_2_O_2_, which determines the time available for H_2_O_2_ generation. To scale up the system for conditions expected in a dry well, the electrode area can be increased by stacking the required number of modular reactors. Finally, a balance between the applied current density, which determines electricity consumption, and the electrode capital cost can be established for each specific application. For example, to treat a typical 8-hour storm event in which water flows at the full infiltration capacity of the drywell (*Q* = 400 L/min) with an initial H_2_O_2_ concentration of 1 mM in the stormwater (detailed discussion about [H_2_O_2_]_initial_ on the treatment performance is described in the following section), the required amount of H_2_O_2_ stock solution ([H_2_O_2_]_stock_ = 200 mM, *V* = 1 m^3^) can be generated with five 40 cm × 40 cm air-diffusion cathodes operating at a current density of 800 A/m^2^ for one day prior to the storm event. The 1 m^3^ H_2_O_2_ stock solution reservoir and the electrochemical modules can be deployed within the available service area next to the drywell.

### Trace organic contaminant removal by advanced oxidation

To assess the performance of the UV/H_2_O_2_ AOP, the rate of transformation of a trace organic contaminant that is lost mainly through reactions with **•**OH (i.e., CBZ) was evaluated in simulated stormwater. Upon exposure to UV light and H_2_O_2_, most trace organic contaminants in stormwater react with **•**OH radical at near diffusion-controlled rates (10^9^–10^10^ M^–1^ s^–1^) (Wols and Hofman-Caris 2012). Some trace organic contaminants may also be transformed by photolysis. For CBZ, the contribution of photolysis to its removal is expected to be negligible relative to its reactions with **•**OH (Text S6).

**Fig. 4:**
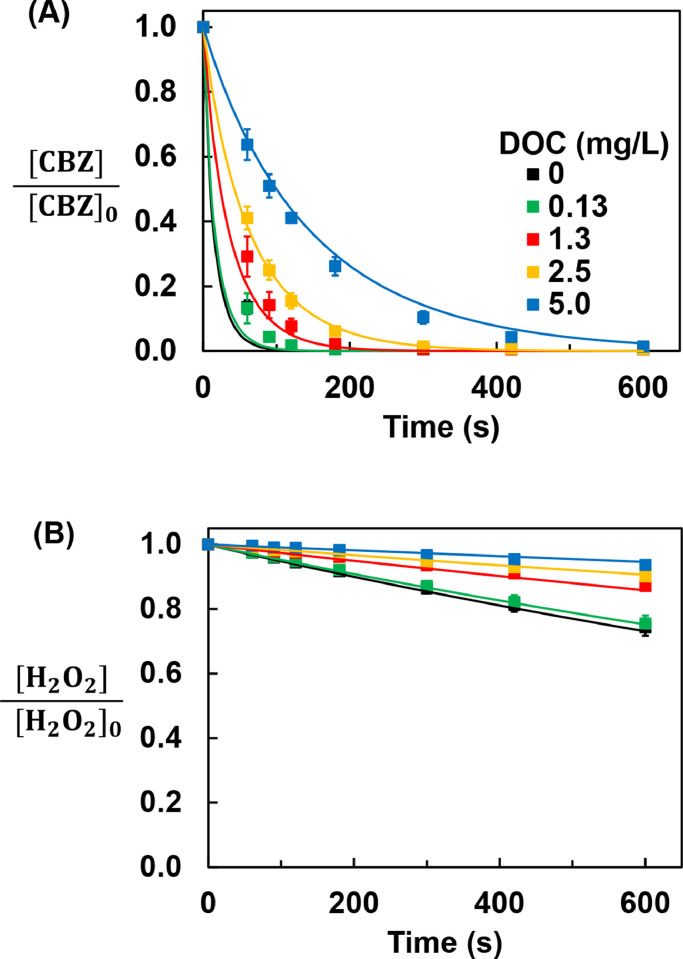
Effect of humic acid concentrations on [H_2_O_2_] and [CBZ] in the AOP. (A) CBZ concentration and (B) H_2_O_2_ concentration. [H_2_O_2_]_initial_ = 1 mM; [CBZ]_initial_ = 10 μg/L. Symbols and lines are assigned to experimental data and model predictions, respectively. Error bars represent one standard deviation; error bars not shown are smaller than symbols.

Measured concentrations of H_2_O_2_ and CBZ were consistent with model predictions (Fig. 4). The modest overprediction of transformation rates at low humic acid concentrations (i.e., 0 and 0.13 mg-C/L) was likely caused by impurities in the deionized water (i.e., dissolved organic carbon concentrations in deionized water can be as high as 0.5 mg-C/L, given that ion-exchange resins do not remove the organics from the tap water, which had a DOC concentration ranging from 1.5 to 3.0 mg-C/L; East Bay Municipal Utility District, 2019). The small underprediction in CBZ removal rate at the highest humic acid concentration (5.0 mg-C/L) was likely caused by the uncertainty of the rate constant for the reaction between **•**OH and humic acid (typically ± 50%) (Appiani et al., 2014). Under conditions representative of stormwater treatment (i.e., [H_2_O_2_]_initial_ and [CBZ]_initial_ of 1 mM and 10 μg/L, respectively), the rate of transformation of the trace organic contaminants decreased as the concentration of humic acid increased. At the highest dissolved organic carbon condition tested (5.0 mg-C/L humic acid), the half-life for CBZ removal was around 1.6 min.

The UV/H_2_O_2_ process should also result in inactivation of pathogens. The photon fluence rates measured by chemical actinometry indicated that the UV dose delivered to water within 10 min of treatment were 2430 mJ/cm^2^, 2300 mJ/cm^2^, 1700 mJ/cm^2^, 1480 mJ/cm^2^ and 1360 mJ/cm^2^ for humic acid concentration ranging from 0 mg-C/L to 5.0 mg-C/L. For reference, 4-log inactivation of *Cryptosporidium, Giardia*, and viruses requires 22 mJ/cm^2^, 22 mJ/cm^2^, and 186 mJ/cm^2^, respectively (USEPA 2003).

To avoid deposition of solutes and minerals on the surface of the UV lamps, the UV reactor was designed with an air gap between the UV lamp and the water surface (Figure S12). Although this design minimizes complications associated with submerged lamps, it decreases light utilization efficiency, because a substantial fraction of the UV light is reflected and backscattered at the air-water interface and converted into heat. Although the aluminum reflector above the lamp redirects some of the light emitted from the lamp and light reflected from the air-water interface back to the water, it only returns about 70% of UV light per reflection (Li 2000). The percentage of UV light lost as heat was calculated as follows:

(8) $$\text{UV}\;\text{light}\;\text{lost}\;\text{as}\;\text{heat}\;\left(\%\right)=1-\frac{W_{254}\;N_{A}\;h\nu \;A\;}{P\;\eta }$$

• Where: W_254_ = Photon fluence rate at 254 nm (Ei cm^–2^ s^–1^)
• N_A_ = 6.02 × 10^23^ mol^–1^
• hν=Energyperphoton (J)
• A = Air-water interface area (cm^2^)
• P = Power of UV lamp (W)
• η = Electrical to UV conversion efficiency, 35% (USEPA 2003)

**Fig. 5:**
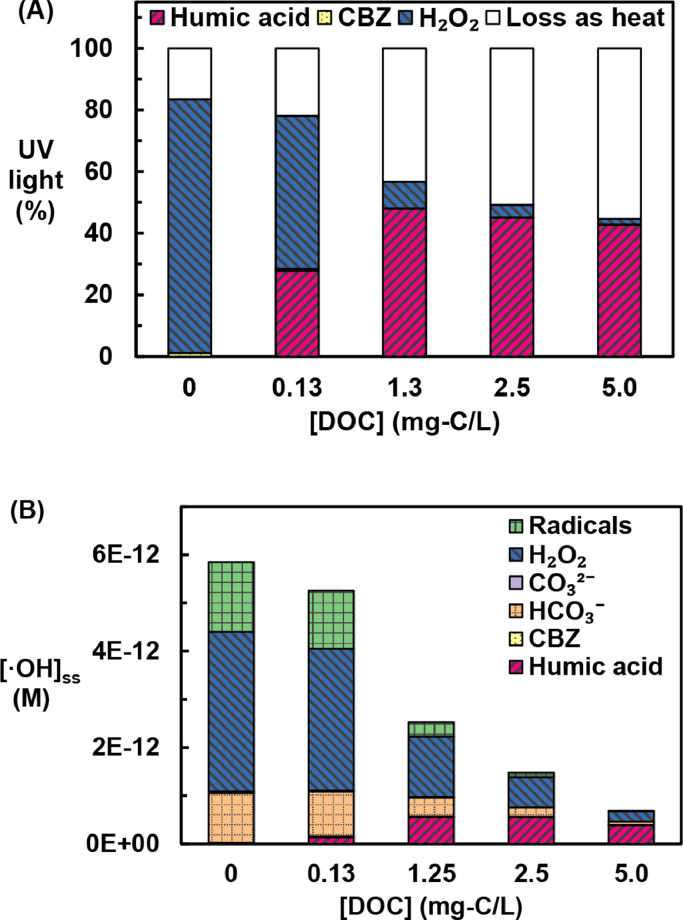
Effect of humic acid concentrations on: (A) UV light loss by reflection and backscattering and through absorption by chromophores; (B) estimated steady-state **•**OH concentration and **•**OH scavenging by scavengers and contaminants (Radicals: O_2_**^–^•**, HO_2_**•**). The height indicates steady state **•**OH concentration and the patterns within indicate the portion of the **•**OH consumed by each species. [H_2_O_2_]_initial_ = 1 mM; [CBZ]_initial_ = 10 μg/L; P_lamp_ = 60 W.

The percentage of UV light lost as heat increased from 17% to 55% as the concentration of humic acid increased from 0 to 5.0 mg-C/L because the humic acid enhanced reflection and backscattering of UV light at the air-water interface (Fig. 5A). The increased light reflection and backscattering with dissolved species agreed with observations from previous research (Clarke, Ewing and Lorenzen, 1970, Grüniger, Möbius and Meyer, 1983; Kozarac et al., 2005; Schwarzenbach et al., 2016). For the light absorbed in the water column, the fraction of light absorbed by each chromophore was calculated based on the concentration and the molar attenuation coefficient. The percentage of UV light absorbed by H_2_O_2_ decreased from 99% to 4% as the concentration of humic acid increased from 0 to 5.0 mg-C/L. As a result, the **•**OH production rate decreased and the rate of H_2_O_2_ loss also decreased.

In addition to slowing the rate of **•**OH production by competing with H_2_O_2_ for UV light, humic acid also slowed the CBZ removal by serving as a **•**OH scavenger (Fig. 5B and Text S7). The concentrations of **•**OH were obtained from the Kintecus kinetic model over experimental time-scales. The steady-state concentrations of **•**OH, which were estimated by the average value of the **•**OH concentrations, decreased by about 90% as the humic acid concentration increased from 0 to 5.0 mg-C/L. As the concentration of humic acid increased, its relative importance as a **•**OH scavenger also increased. At 5.0 mg-C/L of humic acid, close to half of the **•**OH produced was scavenged by humic acid. Because the model did not account for the transition metal catalyzed dismutation of O_2_**^–^•**, the steady-state concentration of this species was probably overestimated O_2_**^–^•**. Therefore, the actual scavenging effect of O_2_**^–^•** (included under “radicals” in Fig. 5B) is probably less important, especially at low humic acid concentrations.

Despite the fact that **•**OH react quickly with humic acid, CBZ was removed from stormwater in the presence of 5.0 mg-C/L of humic acid because the humic acid only reduced the steady-state **•**OH concentration by 90%. However, the overall performance of the system is expected to decrease dramatically as the humic acid concentration further increases due to the inefficient UV reactor design and the scavenging effect. Therefore, under conditions likely to be useful in the field, the maximum humic acid concentration in stormwater treated by this system is around 5 mg-C/L.

### Full-scale treatment system design and considerations

**Fig. 6:**
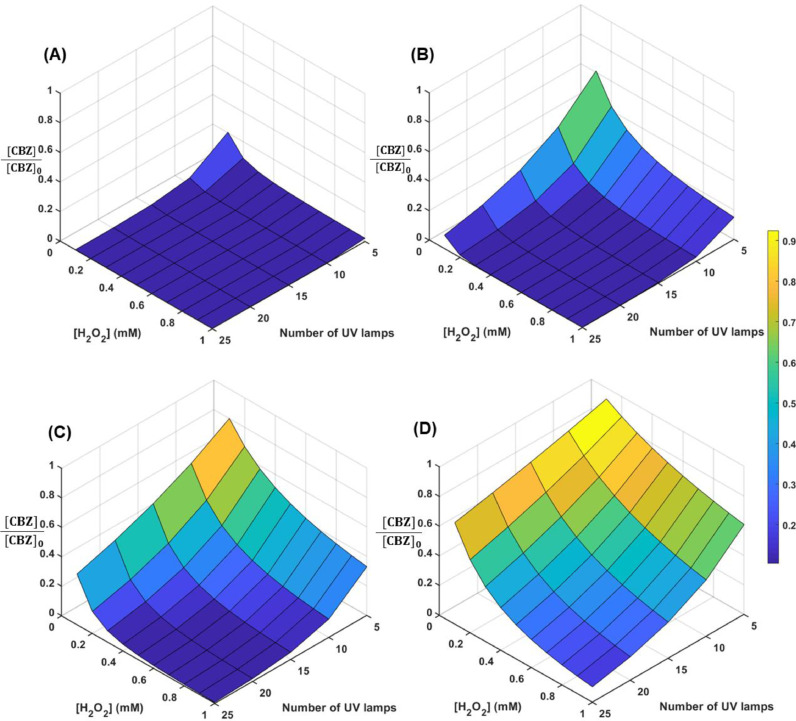
Predicted CBZ removal for operation of the full-scale system under different conditions including: initial [H_2_O_2_], number of 500-W UV lamps, and stormwater containing different concentrations of humic acid (A) 0.13 mg-C/L, (B) 1.25 mg-C/L, (C) 2.5 mg-C/L, and (D) 5.0 mg-C/L.

The kinetic model can provide guidance for the design of full-scale treatment systems and their operation under different conditions. Considering a baseline case with a maximum flow rate of 400 L/min (i.e., 0.067 m^3^/s), which is the typical value for drywells (Torrent Resources, 2021), along with other design conditions (e.g., system size limited by space available to install a treatment system within a drywell), the UV reactor will need to achieve at least 90% removal of a trace organic contaminant at diffusion-controlled rates, if it is transformed mainly by reactions with **•**OH. The initial H_2_O_2_ concentration in stormwater prior to exposure of UV light (hereinafter referred to as “initial H_2_O_2_ concentration” in this section) would need to be at least 0.1 mM; and between 5 and 25 500-watt low-pressure UV lamps would be needed to achieve the treatment goal if the humic acid concentrations ranged from 0.13 mg-C/L to 5.0 mg-C/L (Fig. 6). Considering these constraints, the analysis indicated a trade-off between the initial concentration of H_2_O_2_ and the number of lamps being operated. At a humic acid concentration of 2.5 mg-C/L, 90% of the organic contaminants can be removed with an initial H_2_O_2_ concentration of 0.6 mM and 15 lamps. For a humic acid concentration of 5 mg-C/L, an initial H_2_O_2_ concentration of 1 mM and 25 lamps is needed to assure 90% removal of a trace organic contaminant.

**Fig. 7:**
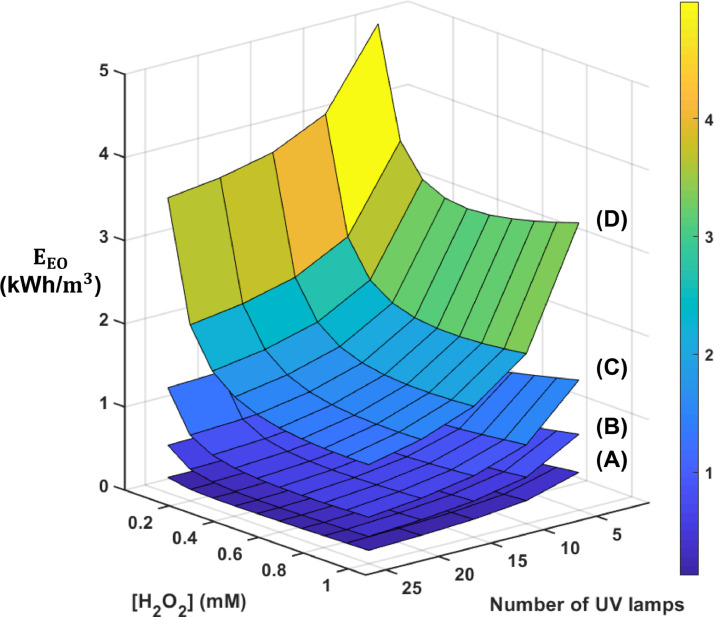
Predicted E_EO_ for operating the full-scale system under different initial [H_2_O_2_], number of UV lamps with stormwater containing different concentrations of humic acid (A) 0.13 mg-C/L, (B) 1.25 mg-C/L, (C) 2.5 mg-C/L, and (D) 5.0 mg-C/L. The power consumption associated with H_2_O_2_ generation was set as 1.9 × 10^−5^ kWh/mg-H_2_O_2_ to represent the average consumption for all current densities.

The initial H_2_O_2_ concentration in stormwater and the number of lamps being used affect the energy consumption by the full-scale UV/H_2_O_2_ system. We used the electrical energy per order (E_EO_) of the system to compare the system performance under different operating conditions (Bolton et al., 2001). Overall, E_EO_ increased with the DOC concentrations because of the light reflection and backscattering at air-water interface, competition for light absorption and **•**OH scavenging by humic acid (Fig. 7). We estimated E_EO_ values of approximate were between 0.5 and 2 kWh/m^3^ for condition that are likely to be employed in full-scale treatment system. For reference, modern seawater desalination requires about 3 to 5 kWh/m^3^ (Kim et al., 2019) and electrochemical oxidation with boron-doped diamond electrodes requires about 40 kWh/m^3^ (Lanzarini-Lopes et al., 2017; Vahid and Khataee 2013).

The specific UV absorbance (SUVA) at 254 nm of humic substances measured in previous study (0.006–0.053 L mg^−1^ cm^−1^) (Weishaar et al., 2003) were lower than the value measured in this study (0.083 ± 0.001 L mg^−1^ cm^−1^). If stormwater with a lower SUVA value at 254 nm than the value used in our model is tested, higher removal of contaminants and lower energy consumption may be observed.

Another factor affecting the energy consumption is the applied current density used when preparing the H_2_O_2_ stock solution. Assuming the power consumption associated with H_2_O_2_ generation was 1.9 × 10^−5^ kWh/mg-H_2_O_2_ (*i* = 600 A/m^2^), the H_2_O_2_ generation process consumed a similar amount of energy as the UV lamp. For example, at 5.0 mg-C/L of humic acid, if the system is operated with an initial H_2_O_2_ concentration of 0.9 mM and 25 UV lamps (500 W), generating enough H_2_O_2_ for one hour of continuous dosing into stormwater would consume about 14 kWh of electricity and operating the lamps for one hour would consume about 13 kWh electricity. Generating the same amount of H_2_O_2_ at current density ranging from 200 A/m^2^ to 1200 A/m^2^ would consume between 7 kWh and 20 kWh of electricity.

Under the conditions employed in the AOP, less than 30% of the H_2_O_2_ will be converted into **•**OH by UV light. Thus, residual H_2_O_2_ (i.e., up to around 1 mM) remaining after the treatment would be recharged to groundwater. Residual H_2_O_2_ should decompose in the first several centimeters of subsurface, mainly through reactions catalyzed by transition metal oxides and microbial biomass (Wang et al., 2016). If necessary, iron-containing minerals or manganese-containing minerals can be added to the drywell to enhance the rate of decomposition of H_2_O_2_ (Pham et al., 2012). Because O_2_ is produced when H_2_O_2_ decomposes through transition metal-catalyzed reactions, this process increases dissolved oxygen concentrations in the subsurface. This is potentially beneficial because aerobic biodegradation of residual organic contaminants (e.g., transformation products formed by the UV/H_2_O_2_ process) tends to be more effective than biotransformation that takes place under anoxic or anaerobic conditions (Lhotský et al., 2017; Mikkonen et al., 2018; Regnery et al., 2017; Ying et al., 2008).

Another consideration for successful operation is maintenance. The suspended UV lamp avoids any potential fouling on the lamp sleeve; therefore, the longevity of the UV reactor is only limited by the lifetime of UV lamps (typically >5000 h). The longevity of the electrochemical reactor is limited by aging of the air-diffusion cathode or the cation exchange membrane. Although additional experiments are needed to further assess the longevity of the air-diffusion cathode, cation exchange membranes have been fabricated to enhance their oxidative stability (they typically operate for >1000 h) (Park et al., 2018). Because the proposed treatment only operates during the rainy season (a total of several hundred hours of operation per year), it appears likely that the proposed system can be operated with only annual maintenance, which is consistent with practices employed by communities that use drywells (Whatcom County, 2021).

## Conclusions

Electrochemical generation of H_2_O_2_ followed by activation of H_2_O_2_ with UV light efficiently removed trace organic contaminants from stormwater. H_2_O_2_ was efficiently generated in stock solutions under different water matrix and applied current densities. The maximum H_2_O_2_ concentrations achieved in the stock solution were between 400 and 600 mM. Although stormwater composition impacted the H_2_O_2_ stock solution stability, neutralization of basic pH conditions by mixing the catholyte and anolyte resulted in sufficient stability to enable H_2_O_2_ generation for up to three days before arrival of a storm and to provide enough H_2_O_2_ to treat a typical storm lasting as long as eight hours.

These lab-scale tests and model predictions, while indicating the potential for removing trace organic contaminants, did not account for several factors that could be encountered in field-scale applications. For example, during H_2_O_2_ generation tests, decreased performance of the electrode was observed after extended use, possibly as a result of flooding (i.e., decreased hydrophobicity of the water-facing side of the electrode). Further research should be conducted to investigate the factors that affect the electrode lifetime (e.g., current density, electrolyte conductivity). Due to the variation in stormwater compositions, H_2_O_2_ decomposition rates in the stock solutions also may merit further assessment. For locations with high concentrations of natural organic matter in stormwater, submerged UV lamps capable of delivering UV light to stormwater more efficiently by avoiding the light reflection and scattering at water/air interface may be needed. However, the tradeoff between lamp fouling and lamp efficiency requires further analysis. UV LED lamps offer promising alternatives because of the minimal heat generation at lamp surface, which reduces the potential for precipitative fouling (Linden et al., 2019). New engineering solutions for fitting the reactors into the drywell may also be advantageous. Low-cost sensors and controllers also may be coupled with operation of this system to maximize water quality benefits and enable remote or automated operations (Kerkez et al., 2016; Mullapudi et al., 2017). Following site-specific optimization, the system should be tested under field conditions.

## Declaration of Competing Interest

The authors declare that they have no known competing financial interests or personal relationships that could have appeared to influence the work reported in this paper.

## Appendix Supplementary materials

Image, application 1
