# Supplementary material for: An electrochemical advanced oxidation process for the treatment of urban stormwater
Source: Water Res X. 2021 Nov 28;13:100127. doi: 10.1016/j.wroa.2021.100127 (PMC8649961; doi:10.1016/j.wroa.2021.100127)
Supplement: Supplementary file 1 [file mmc1.docx]

***Supplementary Material for:***

**An Electrochemical Advanced Oxidation Process for the Treatment of Urban Stormwater**

Yanghua Duan and David L. Sedlak*

Department of Civil & Environmental Engineering, University of California, Berkeley, CA, USA

**29 pages**

**8 tables**

**12 figures**

**References**

^*^Corresponding author e-mail: [sedlak@berkeley.edu](mailto:sedlak@berkeley.edu), T: 510-643-0256

Table of Contents

[Table S1. Simulated stormwater matrix composition. Suwannee River natural organic matter (concentration: 10 mg-C/L) or Sigma humic acid (concentration: 0-5 mg-C/L) was also added to the simulated stormwater matrix in some experiments to examine the effect of dissolved organic carbon on reaction efficiency. S4](#_Toc87983568)

[Text S1. Dissolution of San Joaquin soil in H_2_O_2_ stock solution S4](#_Toc87983569)

[Figure S1. Schematic of the reactor setup for UV/H_2_O_2_ process. S5](#_Toc87983570)

[Table S2. Properties of compounds that commonly detected in stormwater. S6](#_Toc87983571)

[Text S2. Determination of the photon fluence rate S7](#_Toc87983572)

[Figure S2. Natural logarithm of normalized atrazine concentration versus time. [Atrazine]_0_ = 100 μg/L in simulated stormwater with 5 mM phosphate buffer (pH = 7) amended with different concentrations of humic acid. S9](#_Toc87983573)

[Figure S3. Graphically determination of k_real_ value from the actinometry experiments. Symbols and lines are assigned to results calculated based on k_observed_ and k_real_, respectively. S11](#_Toc87983574)

[Table S3. Atrazine photolysis rate constants. S11](#_Toc87983575)

[Table S4. Photon fluence rates for simulated stormwater amended with different concentrations of humic acid (0-5 mg-C/L). S11](#_Toc87983576)

[Text S3. Kinetic model S12](#_Toc87983577)

[Table S5. Reactions relevant to the H_2_O_2_/UV process. S13](#_Toc87983578)

[Table S6. UV absorbance at 254 nm wavelength for chromophores. S14](#_Toc87983579)

[Figure S4. Catholyte pH change during H_2_O_2_ generation. Error bars represent one standard deviation; error bars not shown are smaller than symbols. S14](#_Toc87983580)

[Table S7. Performance metrics of different H_2_O_2_ generation methods. S15](#_Toc87983581)

[Figure S5. Faraday efficiency during electrolysis in 0.2 M Na_2_SO_4_-amended simulated stormwater and 0.2 M Na_2_SO_4_ electrolyte. Error bars represent one standard deviation. S16](#_Toc87983582)

[Text S4. Selection of Na_2_SO_4_ amendment concentration for H_2_O_2_ generation S17](#_Toc87983583)

[Figure S6. Effect of Na_2_SO_4_ amendment concentrations in simulated stormwater and applied current densities on energy consumption for H_2_O_2_ generation. S18](#_Toc87983584)

[Figure S7. Cell voltage as a function of time during electrolysis in 0.2 M Na_2_SO_4_-amended simulated stormwater and 0.2 M Na_2_SO_4_ electrolyte. Error bars represent one standard deviation. S18](#_Toc87983585)

[Text S5. Estimation of the cost for H_2_O_2_ generation S19](#_Toc87983586)

[Figure S8. Estimated salt amendment cost, electricity cost and overall cost for H_2_O_2_ generation at various Na_2_SO_4_ amendment concentrations and current densities; [H_2_O_2_] = 450 mM. S19](#_Toc87983587)

[Figure S9. Energy consumption for 400 mM of H_2_O_2_ solution as a function of current density. Error bars represent one standard deviation; error bars not shown are smaller than symbols. S20](#_Toc87983588)

[Figure S10. Hydrogen peroxide stability in catholyte and catholyte mixed with anolyte. S21](#_Toc87983589)

[Figure S11. Solution pH during H_2_O_2_ storage. Error bars represent one standard deviation; error bars not shown are smaller than symbols. S21](#_Toc87983590)

[Table S8. Concentrations of transition metals measured in the waters used in this study. S22](#_Toc87983591)

[Text S6. Estimation of the contribution of photolysis to carbamazepine removal S23](#_Toc87983592)

[Figure S12. Schematic of light field in the UV reactor. S24](#_Toc87983593)

[Text S7. Fraction of ·OH that reacted with the contaminant or ·OH scavengers S24](#_Toc87983594)

[References S25](#_Toc87983595)

# Table S1. Simulated stormwater matrix composition. Suwannee River natural organic matter (concentration: 10 mg-C/L) or Sigma humic acid (concentration: 0-5 mg-C/L) was also added to the simulated stormwater matrix in some experiments to examine the effect of dissolved organic carbon on reaction efficiency.

| **Constituent** | **Concentration** | **Unit** |
| --- | --- | --- |
| Ca^2+^ | 0.75 | mM |
| Mg^2+^ | 0.075 | mM |
| Na^+^ | 1.75 | mM |
| NH_4_^+^ | 0.072 | mM |
| SO_4_^2−^ | 0.33 | mM |
| HCO_3_^−^ | 1.00 | mM |
| Cl^−^ | 1.70 | mM |
| NO_3_^−^ | 0.072 | mM |
| H_2_PO_4_^−^ | 0.016 | mM |
| Ionic strength | 4.60 | mM |
| pH | 7.46 ± 0.20  6.84 ± 0.30 | (Natural organic matter amended) (Humic acid amended) |

# Text S1. Dissolution of San Joaquin soil in H_2_O_2_ stock solution

The San Joaquin soil was prepared as a 1.00 g/L of slurry in Milli-Q water by placing it in a sonication bath for 30 minutes. A 1-mL aliquot of the soil slurry was transferred to a borosilicate vial after 30 seconds of mixing by a vortex mixer. The soil slurry was then evaporated at 105 ℃ for 2 hours. Finally, the soil was redissolved in 20 mL aliquots of H_2_O_2_ solution.

# Figure S1. Schematic of the reactor setup for UV/H_2_O_2_ process.

# Table S2. Properties of compounds that commonly detected in stormwater.

| Contaminant  group | Compound | Ɛ_254_  (M^−1^ cm^−1^) | Φ_254_ | k**_·_**_OH_  (M^−1^ s^−1^) | Reference |
| --- | --- | --- | --- | --- | --- |
| Pharmaceuticals | Carbamazepine | 6.0×10^3^ | 6.0×10^−4^ | 9.1×10^9^ | (Jasper and Sedlak 2013, Pereira et al. 2007, Vogna et al. 2004) |
| Biocides | Carbendazim | 4.5×10^3^ | 2.3×10^−3^ | 2.2×10^9^ | (Mazellier et al. 2003, Mazellier et al. 2002) |
| Insecticides | Dimethoate | 0^a^ | n.d.^b^ | 8.5×10^9^ | (Marin et al. 2011, Wu et al. 2021) |
|  | Diazinon | 2.0×10^3^ | 8.6×10^−2^ | 9.0×10^9^ | (Shemer and Linden 2006) |
|  | Fipronil | n.d. | n.d. | 3.9×10^9^ | (King et al. 2020) |
|  | Imidacloprid | 1.1×10^4^ | 8.5×10^−3^ | 1.0×10^10^ | (Aregahegn et al. 2017, Armbrust 2000) |
| Herbicides | 2,4-D | 1.5×10^2^ | 9.5×10^−3^ | 5.1×10^9^ | (Benitez et al. 2004, Feigenbrugel et al. 2006) |
|  | Atrazine | 3.9×10^3^ | 4.6×10^−2^ | 3.0×10^9^ | (Acero et al. 2000, Hessler et al. 1993, Nick et al. 1992) |
|  | Dicamba | 4.3×10^2^ | 9.5×10^−2^ | 1.3×10^9^ | (Armbrust 2000, Wong and Chu 2003) |
|  | Diuron | 1.5×10^4^ | 2.0×10^−2^ | 9.5×10^9^ | (Jirkovský et al. 1997, Olasehinde et al. 2013) |
|  | Isoproturon | 5.9×10^3^ | 2.0×10^−3^ | 5.2×10^9^ | (Benitez et al. 2006, Sanches et al. 2010) |
|  | Mecoprop | 2.1×10^2^ | 8.8×10^−1^ | 2.5×10^9^ | (Armbrust 2000, Semitsoglou-Tsiapou et al. 2016) |
|  | Metolachlor | 5.0×10^2^ | 3.0×10^−1^ | 9.1×10^9^ | (Wu et al. 2007) |
| Plasticizers | BPA | 7.5×10^2^ | 4.6×10^−3^ | 6.9×10^9^ | (Baeza and Knappe 2011, Peller et al. 2009) |

^a^absorption of 254 nm UV is insignificant.

^b^n.d. means no data available.

# Text S2. Determination of the photon fluence rate

Photon fluence rates at 254 nm (i.e., the main wavelength of energy for the low-pressure Hg lamp used in the reactor) were determined using 100 μg/L of atrazine (Ɛ_254_ = 3860 M^−1^ cm^−1^, Φ_254_ = 0.046 mol Ei^−1^) as a chemical actinometer (Bolton and Linden 2003, Canonica et al. 2008). The photon fluence rates were characterized for all simulated stormwater compositions tested in the AOP experiments ([humic acid] = 0-5 mg-C/L). The simulated stormwater was amended with 5 mM of phosphate buffer to maintain a pH value of 7. The actinometry experiments were conducted in batch mode by circulating the solution between the UV reactor and a solution reservoir as described in Section 2.5. The fluence was calculated from the phototransformation rate of atrazine:

$-\frac{\mathrm{dC}}{\mathrm{dt}}$= [$\frac{W_{\lambda}}{Z}$ (1-${10}^{{-\alpha}_{\lambda}Z}$) $\frac{Ɛ_{\lambda}\Phi_{\lambda}}{\alpha_{\lambda}}$][C] (S1)

This yields a first-order direct photolysis rate constant, k:

$k=\frac{W_{\lambda}}{Z}$ (1-${10}^{{-\alpha}_{\lambda}Z}$) $\frac{Ɛ_{\lambda}\Phi_{\lambda}}{\alpha_{\lambda}}$ (S2)

where: $W_{\lambda}-$Fluence [$\frac{mE_{i}}{\mathrm{cm}^{2}s}$]

Z$-$Pathlength [$\mathrm{cm}$]

$Ɛ_{\lambda}-$Molar attenuation coefficient [$M^{-1}\mathrm{cm}^{-1}$]

$\alpha_{\lambda}-$ Beam attenuation coefficient [$\mathrm{cm}^{-1}$] =$\sum(Ɛ_{\lambda}C_{i})$

$\Phi_{\lambda}-$Quantum yield

The light pathlength was estimated by dividing the water stream cross-sectional area in the reactor (80 cm^2^) by the width of the air-water interface (30 cm).

By plotting the natural logarithm of atrazine removal with time (Figure S2), the observed photolysis rate constants, k_observed_ (Table S3) were obtained and used to calculate the observed fluence value, W_254, observed_ (Table S4) using:

$W_{\lambda}=\frac{kZ\alpha_{\lambda}}{(1-{10}^{{-\alpha}_{\lambda}Z})Ɛ_{\lambda}\Phi_{\lambda}}$ (S3)

# Figure S2. Natural logarithm of normalized atrazine concentration versus time. [Atrazine]_0_ = 100 μg/L in simulated stormwater with 5 mM phosphate buffer (pH = 7) amended with different concentrations of humic acid.

Mass balance was applied to deconvolute the effect of water recirculation on determination of the photolysis rate constants (k_real_) and photon fluence rates (W_254, real_). The solution reservoir was assumed to act as a continuous stirred-tank reactor with no reaction; The UV reactor was simplified as a plug-flow reactor without dispersion (Figure S1).

Within one hydraulic residence time of the UV reactor (HRT_UV_), the effluent concentration of atrazine was not related to the feed water but depended only on the time of exposure to UV light, which was determined by the reaction time, and the initial atrazine concentration. After one HRT_UV_, the water fed from the solution reservoir started to flow out of the UV reactor, and water that flowing through the UV reactor was exposed to UV light for one HRT_UV_ of duration. Therefore, the effluent concentration of the UV reactor, C_1_, can be expressed as:

$C_{1}(t)=\left\{ \begin{aligned} C_{0}\exp\left( -k_{\mathrm{real}}t \right) t\leq\mathrm{HRT}_{\mathrm{UV}} \\ C_{2}(t)\exp\left( -k_{\mathrm{real}}\mathrm{HRT}_{\mathrm{UV}} \right) t>\mathrm{HRT}_{\mathrm{UV}} \end{aligned} \right.$ (S4)

For the concentration inside the solution reservoir, C:

$V_{1}\frac{\mathrm{dC}}{\mathrm{dt}}=QC_{1}-QC_{2}=$ $QC_{1}-QC$ (S5)

Initial condition: C = C_0_

where C_0_, C_1_, C_2_, are initial atrazine concentration, atrazine concentration in the effluent and influent of the UV reactor. V_1_ and V_2_ are the volume of the solution reservoir and UV reactor, respectively.

The k_real_ value is determined as the value that led to the minimal sum of squared residuals of the C/C_0_ values after one HRT_UV_ (Table S3). The results were plotted in Figure S3. The W_254, real_ was calculated based on eq S3 (Table S4).

# Figure S3. Graphically determination of k_real_ value from the actinometry experiments. Symbols and lines are assigned to results calculated based on k_observed_ and k_real_, respectively.

# Table S3. Atrazine photolysis rate constants.

| [Humic acid]  (mg-C/L) | 0 | 0.13 | 1.3 | 2.5 | 5.0 |
| --- | --- | --- | --- | --- | --- |
| k_observed_ (s^−1^) | 3.5×10^−3^ | 3.2×10^−3^ | 1.8×10^−3^ | 1.2×10^−3^ | 7.0×10^−4^ |
| k_real_ (s^−1^) | 7.4×10^−3^ | 6.7×10^−3^ | 3.7×10^−3^ | 2.5×10^−3^ | 1.4×10^−3^ |

# Table S4. Photon fluence rates for simulated stormwater amended with different concentrations of humic acid (0-5 mg-C/L).

| [Humic acid]  (mg-C/L) | 0 | 0.13 | 1.3 | 2.5 | 5.0 |
| --- | --- | --- | --- | --- | --- |
| W_254, observed_  (mEi/cm^2^/s) | 8.6×10^−6^ | 8.1×10^−6^ | 6.0×10^−6^ | 5.3×10^−6^ | 4.8×10^−6^ |
| W_254, real_  (mEi/cm^2^/s) | 1.8×10^−5^ | 1.7×10^−5^ | 1.2×10^−5^ | 1.1×10^−5^ | 9.7×10^−6^ |

# Text S3. Kinetic model

Previously published studies have described the reaction mechanisms in the UV/H_2_O_2_ process and measurement of rate constants for the reactions involved in the transformation of trace organic compounds (Baxendale and Wilson 1957, Crittenden et al. 1999, Glaze et al. 1995). Under UV irradiation, each molecule of H_2_O_2_/ HO_2_^−^ is cleaved to form two **·**OH or one **·**OH and one **·**O^−^, which react with H_2_O to produce **·**OH. A substantial fraction of **·**OH radicals react with H_2_O_2_/ HO_2_^−^ to initiate a chain reaction that results in further decomposition of H_2_O_2_ (Table S5 reaction 4, 5, 8, 9, 11, 13, 14). Some of the **·**OH also react with the trace organic compound, humic substances or HCO_3_^−^.

The photolysis rate of H_2_O_2_ is governed by the rate of reaction 1 (Table S5). The quantum yield of the primary process (reaction 1) $\Phi_{H_{2}O_{2},primary}$ at 254 nm is 0.5 (Baxendale and Wilson 1957). However, the overall quantum yield $\Phi_{H_{2}O_{2,overall}}$, which describes the overall process including the chain reactions is unity at 254 nm (Baxendale and Wilson 1957).

# Table S5. Reactions relevant to the H_2_O_2_/UV process.

|  | Reaction | Reaction rate constant | Reference |
| --- | --- | --- | --- |
| 1 | H_2_O_2_$\underset{\to}{\mathrm{hv}}$ 2**·**OH | k_1_ $=\frac{W_{\lambda}}{Z}$ (1-${10}^{{-\alpha}_{\lambda}Z}$) $\frac{Ɛ_{H_{2}O_{2}}\Phi_{H_{2}O_{2}}}{\alpha_{\lambda}}$ |  |
| 2 | HO_2_^−^ + H_2_O$\underset{\to}{\mathrm{hv}}$ 2**·**OH + OH^−^ | k_2_ $=\frac{W_{\lambda}}{Z}$ (1-${10}^{{-\alpha}_{\lambda}Z}$) $\frac{Ɛ_{\mathrm{HO}_{2}^{-}}\Phi_{\mathrm{HO}_{2}^{-}}}{\alpha_{\lambda}}$ |  |
| 3 | CBZ$\underset{\to}{\mathrm{hv}}$ ? | k_3_ $=\frac{W_{\lambda}}{Z}$ (1-${10}^{{-\alpha}_{\lambda}Z}$) $\frac{Ɛ_{\mathrm{CBZ}}\Phi_{\mathrm{CBZ}}}{\alpha_{\lambda}}$ |  |
| 4 | **·**OH + H_2_O_2_ $\underset{\to}{}$O_2_^•−^ + H_2_O + H^+^ | k_4_ = 2.7 × 10^7^ M^−1^ s^−1^ | (Buxton et al. 1988) |
| 5 | **·**OH + HO_2_^−^ $\underset{\to}{}$O_2_^•−^+ H_2_O | k_5_ = 7.5 × 10^9^ M^−1^ s^−1^ | (Buxton et al. 1988) |
| 6 | **·**OH + HCO_3_^−^ $\underset{\to}{}$H_2_O + **·**CO_3_^−^ | k_6_ = 8.5 × 10^6^ M^−1^ s^−1^ | (Buxton et al. 1988) |
| 7 | **·**OH + CO_3_^2−^ $\underset{\to}{}$ OH^−^ + **·**CO_3_^−^ | k_7_ = 3.9 × 10^8^ M^−1^ s^−1^ | (Buxton et al. 1988) |
| 8 | **·**OH + HO_2_**·** $\underset{\to}{}$H_2_O + O_2_ | k_8_ = 8.0 × 10^9^ M^−1^ s^−1^ | (Elliot and Buxton 1992, Sehested et al. 1968) |
| 9 | **·**OH + O_2_^•−^$\underset{\to}{}$ OH^−^ + O_2_ | k_9_ = 6.6 × 10^9^ M^−1^ s^−1^ | (Sehested et al. 1968) |
| 10 | **·**OH + CBZ $\underset{\to}{} ?$ | k_CBZ,_**_·_**_OH_ = 9.1 × 10^9^ M^−1^ s^−1^ | (Jasper and Sedlak 2013) |
| 11 | O_2_^•−^ + H_2_O_2_ $\underset{\to}{}$ **·**OH + OH^−^ + O_2_ | k_11_ = 0.13 M^−1^ s^−1^ | (Weinstein and Bielski 1979) |
| 12 | O_2_^•−^ + **·**CO_3_^−^ $\underset{\to}{}$ O_2_ + CO_3_^2−^ | k_12_ = 6.5 × 10^8^ M^−1^ s^−1^ | (Eriksen et al. 1985) |
| 13 | O_2_^•−^ + HO_2_**·** + H_2_O $\underset{\to}{}$ H_2_O_2_ + $O$H^−^ + O_2_ | k_13_ = 9.7 × 10^7^ M^−1^ s^−1^ | (Buxton et al. 1988) |
| 14 | HO_2_**·** + HO_2_**·** $\underset{\to}{}$ H_2_O_2_ + O_2_ | k_14_ = 8.3 × 10^5^ M^−1^ s^−1^ | (Buxton et al. 1988) |
| 15 | **·**CO_3_^−^ + H_2_O_2_ $\underset{\to}{}$ HCO_3_^−^ + O_2_^•−^ + H^+^ | k_15_ = 8.0 × 10^5^ M^−1^ s^−1^ | (Draganić et al. 1991) |
| 16 | **·**CO_3_^−^ + HO_2_^−^ $\underset{\to}{}$ HCO_3_^−^ + O_2_^•−^ | k_16_ = 3.0 × 10^7^ M^−1^ s^−1^ | (Draganić et al. 1991) |
| 17 | **·**CO_3_^−^ + **·**CO_3_^−^ $\underset{\to}{}$? | k_17_ = 3.0 × 10^7^ M^−1^ s^−1^ | (Huie and Clifton 1990, Mandal et al. 1991) |
| 18 | **·**OH + DOC $\underset{\to}{} ?$ | k_18_ = 9.8 × 10^3^ L mgC^−1^ s^−1^ | (Appiani et al. 2014) (Barazesh et al. 2015) |
| 19 | **·**OH + Cl^−^ $\underset{\to}{}$ HOCl**^·^**^−^ | k_19_ = 4.3 × 10^9^ M^−1^ s^−1^ | (Jayson et al. 1973) |
| 20 | HOCl**^·^**^−^ $\underset{\to}{}$ **·**OH + Cl^−^ | k_20_ = 6.1 × 10^9^ s^−1^ | (Jayson et al. 1973) |
| 21 | HOCl**^·^**^−^ + H^+^ $\underset{\to}{}$ Cl**·**+ H_2_O | k_21_ = 2.1 × 10^10^ M^−1^ s^−1^ | (Jayson et al. 1973) |
| 22 | **·**CO_3_^−^ + CBZ$\underset{\to}{} ?$ | k_22_ = 2.3 × 10^6^ M^−1^ s^−1^ | (Jasper and Sedlak 2013) |

The molar absorption coefficients of the major chromophores are summarized in Table S6. The molar absorption coefficient for humic acid at 254 nm was experimentally measured using a Shimadzu UV-2600 spectrophotometer and a Shimadzu TOC-V analyzer.

# Table S6. UV absorbance at 254 nm wavelength for chromophores.

| Species | DOC | Carbamazepine | H_2_O_2_ |
| --- | --- | --- | --- |
| ε | 0.083 ± 0.001 (L mg^−1^ cm^−1^) | 6025 (M^−1^ cm^−1^) | 18.6 (M^−1^ cm^−1^) |
| References | This study | (Vogna et al. 2004) | (Morgan et al. 1988) |

The observed photon fluences, W_254, observed_, were used for model validation. For full-scale performance prediction, the UV fluence rates were calculated with number of UV lamps (N), power of UV lamps (P, W) and volume of the full-scale reactor (V_full-scale_, L) and lab-scale reactor (V_UV_, L), and W_254, real_:

$W_{full-scale}=\frac{N\cdot P\cdot W_{254,real}\cdot V_{\mathrm{UV}}}{60W V_{full-scale}}$ (S6)

# Figure S4. Catholyte pH change during H_2_O_2_ generation. Error bars represent one standard deviation; error bars not shown are smaller than symbols.

# Table S7. Performance metrics of different H_2_O_2_ generation methods.

| Cathodic catalyst | Electrolyte | Production rate (mmol cm^−2^ hour^−1^) | Faraday  efficiency (%) | [H_2_O_2_]_max_  (mM) | Reference |
| --- | --- | --- | --- | --- | --- |
| Graphite particles | 2 M NaOH | N/A | 60 | 800 | (Oloman and Watkinson 1979) |
| Carbon black and PTFE | 0.2 M Na_2_SO_4_ | 0.6 | 21 | 320 | (Luo et al. 2015) |
| Activated carbon, vapor-growing-carbon fiber, and PTFE | 1.2 N H_2_SO_4_ | 0.14 | 40 | 350 | (Yamanaka et al. 2008) |
| Activated carbon, vapor-growing-carbon fiber, and PTFE | Nafion 117 | 0.04 | 12 | 950 | (Yamanaka and Murayama 2008) |
| Heat-treated Mn–porphyrin/Carbon | 0.5 M H_2_SO_4_ | 0.4 | 47 | 1100 | (Yamanaka et al. 2006) |
| CoTPP/Ketjen-Black | Nafion-H | 0.9 | 55 | 5500 | (Iwasaki et al. 2018) |
| Mesoporous carbon (CMK-3) | 0.1 M KOH | N/A | >90 | 80 | (Chen et al. 2017) |
| Edge site-rich nanocarbon | 0.1 M KOH | N/A | 99 | 24 | (Sa et al. 2019) |
| Carbon-supported Ni^II^ single-atom catalysts with  a tetradentate Ni-N_2_O_2_ coordination | 0.1 M KOH | 1.2 | >90 | 250 | (Wang et al. 2020) |
| Co–C composite catalyst | Nafion 112 | 0.2 | 30 | N/A | (Li et al. 2013) |
| Al_2_O_3_/platinum diphosphide nanocrystals | Polymer electrolyte | 2.3 | 79 | 980 | (Li et al. 2020) |
| Functionalized carbon black | Solid electrolyte | 3.4 | 90-95 | 5900 | (Xia et al. 2019) |
| Black carbon | As described in method | 0.3-1.6 | 60-98* | 600 | This study |

* Faraday efficiency decreased during electrolysis due to H_2_O_2_ decomposition (Figure S5)

# Figure S5. Faraday efficiency during electrolysis in 0.2 M Na_2_SO_4_-amended simulated stormwater and 0.2 M Na_2_SO_4_ electrolyte. Error bars represent one standard deviation.

# Text S4. Selection of Na_2_SO_4_ amendment concentration for H_2_O_2_ generation

To determine the optimal salt dosage for H_2_O_2_ generation, electrolysis was performed in simulated stormwater (10 mg-C/L of NOM) amended with varying concentrations of Na_2_SO_4_ (Figure S6). The energy consumption for H_2_O_2_ generation was estimated assuming a Faraday efficiency $\eta$ = 65 % and calculated based on the cell voltage recorded after at least one hour of electrolysis:

$\mathrm{Energy}\left( \frac{\mathrm{kWh}}{\mathrm{mg} H_{2}O_{2}} \right)= \frac{iV_{\mathrm{cell}}\mathrm{At}}{\frac{\mathrm{iAt}}{2F}\cdot\eta}=\frac{2FV_{\mathrm{cell}}}{\eta}\frac{1 mol}{34000 mgH_{2}O_{2}}\frac{1 kWh}{3.6\times{10}^{6} J}$ (S7)

Where, i = Current density (A/m^2^)

V_cell_ = Cell voltage (V)

A = Electrode area (m^2^)

t = Electrolysis time (s)

F = 96485 (C/mol)

The cell voltage after one hour of electrolysis was used because it can be used as a rough approximation of the averaged cell voltage through the whole H_2_O_2_ generation process (Figure S7).

# Figure S6. Effect of Na_2_SO_4_ amendment concentrations in simulated stormwater and applied current densities on energy consumption for H_2_O_2_ generation.

# Figure S7. Cell voltage as a function of time during electrolysis in 0.2 M Na_2_SO_4_-amended simulated stormwater and 0.2 M Na_2_SO_4_ electrolyte. Error bars represent one standard deviation.

# Text S5. Estimation of the cost for H_2_O_2_ generation

The salt amendment cost, electricity cost and overall cost to generate a 450 mM H_2_O_2_ stock solution were estimated at various Na_2_SO_4_ amendment concentrations and current densities (Figure S8). The cost of Na_2_SO_4_ was assumed as $100/ton. The electricity cost was assumed as the average price of electricity in California, $0.16/kWh (U.S. Energy Information Administration 2020).

# Figure S8. Estimated salt amendment cost, electricity cost and overall cost for H_2_O_2_ generation at various Na_2_SO_4_ amendment concentrations and current densities; [H_2_O_2_] = 450 mM.

# Figure S9. Energy consumption for 400 mM of H_2_O_2_ solution as a function of current density. Error bars represent one standard deviation; error bars not shown are smaller than symbols.

# **Figure S10.** Hydrogen peroxide stability in catholyte and catholyte mixed with anolyte.

# Figure S11. Solution pH during H_2_O_2_ storage. Error bars represent one standard deviation; error bars not shown are smaller than symbols.

# Table S8. Concentrations of transition metals measured in the waters used in this study.

| Concentration  (μg/L) | 0.2 M Na_2_SO_4_ electrolyte | 0.2 M Na_2_SO_4_-amended stormwater |
| --- | --- | --- |
| Cr | 3.2 ± 1.0 | 3.2 ± 0.04 |
| Mn | 1.2 ± 0.51 | 1.1 ± 0.07 |
| Fe | 3.4 ± 2.2 | 24.1 ± 0.57 |
| Ni | <3.5 | <3.5 |
| Cu | 0.3 ± 0.06 | 2.1 ± 0.04 |
| Zn | 120 ± 8 | 150 ± 2 |

# Text S6. Estimation of the contribution of photolysis to carbamazepine removal

The direct photolysis did not contribute significantly to the degradation of carbamazepine during the UV/H_2_O_2_ process because of its low quantum yield, 6.0×10^−4^ (Pereira et al. 2007). Based on previously study (Ko et al. 2009), the effect of nitrate on contaminant removal during the UV/H_2_O_2_ process was negligible at initial H_2_O_2_ concentration above 20 mg/L. The contribution of indirect photolysis by photo-excited dissolved organic matter (DOC*) for carbamazepine removal was also negligible (Lee et al. 2014). No data is available for the reaction between carbamazepine with ^1^O_2_.

The formation rate R_form_ (M s^−1^) of **·**OH from DOM and H_2_O_2_ were calculated from the rate of H_2_O_2_ photolysis:

R_form_ = $n_{\boldsymbol{\cdot}\mathrm{OH}}$[$\frac{W_{\lambda}}{Z}$ (1-${10}^{{-\alpha}_{\lambda}Z}$) $\frac{Ɛ_{\lambda}\Phi_{\lambda}}{\alpha_{\lambda}}$][C] (S8)

Where, $n_{\boldsymbol{\cdot}\mathrm{OH}}$ = mole of **·**OH formed per mole of reaction.

The ratio of $R_{form, DOM}$to $R_{form, H_{2}O_{2}}$ in the condition of 5 mg-C/L of humic acid in stormwater can be calculated as:

$\frac{R_{form, DOM}}{R_{form, H_{2}O_{2}}}=\frac{{n_{\boldsymbol{\cdot}\mathrm{OH}} Ɛ}_{\mathrm{DOM}}\Phi_{\mathrm{DOM}} \left[ \mathrm{DOM} \right]}{{n_{\boldsymbol{\cdot}\mathrm{OH}} Ɛ}_{H_{2}O_{2}}\Phi_{H_{2}O_{2}} \left[ H_{2}O_{2} \right]}$ (S9)

$=\frac{0.017 \left( L \mathrm{mg}^{-1}cm^{-1} \right)\times3.7\times{10}^{-5} \times5 (mg L^{-1})}{2\times18.6 \left( M^{-1}cm^{-1} \right)\times0.5 \times{10}^{-3} (M)}=1.7\times{10}^{-4}$

The quantum yield $\Phi_{\mathrm{DOM}}$ was adapted from Jasper and Sedlak (2013).

# Figure S12. Schematic of light field in the UV reactor.

# Text S7. Fraction of ·OH that reacted with the contaminant or ·OH scavengers

The fraction of **·**OH that reacted with the contaminant or a specific **·**OH scavenger was estimated as:

$Fraction of\boldsymbol{\cdot}OH to species i= \frac{k_{\boldsymbol{\cdot}OH, i}C_{i}}{\sum k_{\boldsymbol{\cdot}\mathrm{OH}} C}$ (S10)

Where: k**_·_**_OH_ = second order reaction rate constants of species i with **·**OH (M^−1^ s^−1^)

C = Concentration of species that react with **·**OH (M)

The scavenging effect of Cl^−^ was neglected because of the fast kinetics of the reverse reaction (Reaction 19 in Table S5).

# References

Acero, J.L., Stemmler, K. and von Gunten, U. (2000) Degradation Kinetics of Atrazine and Its Degradation Products with Ozone and OH Radicals:  A Predictive Tool for Drinking Water Treatment. Environmental Science & Technology 34(4), 591-597.

Appiani, E., Page, S.E. and McNeill, K. (2014) On the use of hydroxyl radical kinetics to assess the number-average molecular weight of dissolved organic matter. Environmental science & technology 48(20), 11794-11802.

Aregahegn, K.Z., Shemesh, D., Gerber, R.B. and Finlayson-Pitts, B.J. (2017) Photochemistry of Thin Solid Films of the Neonicotinoid Imidacloprid on Surfaces. Environmental Science & Technology 51(5), 2660-2668.

Armbrust, K.L. (2000) Pesticide hydroxyl radical rate constants: Measurements and estimates of their importance in aquatic environments. Environmental Toxicology and Chemistry 19(9), 2175-2180.

Baeza, C. and Knappe, D.R.U. (2011) Transformation kinetics of biochemically active compounds in low-pressure UV Photolysis and UV/H_2_O_2_ advanced oxidation processes. Water research 45(15), 4531-4543.

Barazesh, J.M., Hennebel, T., Jasper, J.T. and Sedlak, D.L. (2015) Modular advanced oxidation process enabled by cathodic hydrogen peroxide production. Environmental science & technology 49(12), 7391-7399.

Baxendale, J. and Wilson, J. (1957) The photolysis of hydrogen peroxide at high light intensities. Transactions of the Faraday Society 53, 344-356.

Benitez, F.J., Acero, J.L., Real, F.J. and Roman, S. (2004) Oxidation of MCPA and 2,4-d by UV Radiation, Ozone, and the Combinations UV/H_2_O_2_ and O_3_/H_2_O_2_. Journal of Environmental Science and Health, Part B 39(3), 393-409.

Benitez, F.J., Real, F.J., Acero, J.L. and Garcia, C. (2006) Photochemical oxidation processes for the elimination of phenyl-urea herbicides in waters. Journal of hazardous materials 138(2), 278-287.

Bolton, J.R. and Linden, K.G. (2003) Standardization of methods for fluence (UV dose) determination in bench-scale UV experiments. Journal of environmental engineering 129(3), 209-215.

Buxton, G.V., Greenstock, C.L., Helman, W.P. and Ross, A.B. (1988) Critical review of rate constants for reactions of hydrated electrons, hydrogen atoms and hydroxyl radicals (⋅OH/⋅O^−^ in aqueous solution. Journal of physical and chemical reference data 17(2), 513-886.

Canonica, S., Meunier, L. and Von Gunten, U. (2008) Phototransformation of selected pharmaceuticals during UV treatment of drinking water. Water research 42(1-2), 121-128.

Chen, Z., Chen, S., Siahrostami, S., Chakthranont, P., Hahn, C., Nordlund, D., Dimosthenis, S., Nørskov, J.K., Bao, Z. and Jaramillo, T.F. (2017) Development of a reactor with carbon catalysts for modular-scale, low-cost electrochemical generation of H_2_O_2_. Reaction Chemistry & Engineering 2(2), 239-245.

Crittenden, J.C., Hu, S., Hand, D.W. and Green, S.A. (1999) A kinetic model for H_2_O_2_/UV process in a completely mixed batch reactor. Water research 33(10), 2315-2328.

Draganić, Z.D., Negron-Mendoza, A., Sehested, K., Vujošević, S.I., Navarro-Gonzales, R., Albarran-Sanchez, M. and Draganić, I.G. (1991) Radiolysis of aqueous solutions of ammonium bicarbonate over a large dose range. International Journal of Radiation Applications and Instrumentation. Part C. Radiation Physics and Chemistry 38(3), 317-321.

Elliot, A.J. and Buxton, G.V. (1992) Temperature dependence of the reactions OH + O^–^_2_ and OH + HO_2_ in water up to 200° C. Journal of the Chemical Society, Faraday Transactions 88(17), 2465-2470.

Eriksen, T., Lind, J. and Merenyi, G. (1985) On the acid-base equilibrium of the carbonate radical. Radiation Physics and Chemistry (1977) 26(2), 197-199.

Feigenbrugel, V., Le Calvé, S. and Mirabel, P. (2006) Molar absorptivities of 2,4-D, cymoxanil, fenpropidin, isoproturon and pyrimethanil in aqueous solution in the near-UV. Spectrochimica Acta Part A: Molecular and Biomolecular Spectroscopy 63(1), 103-110.

Glaze, W.H., Lay, Y. and Kang, J.-W. (1995) Advanced oxidation processes. A kinetic model for the oxidation of 1, 2-dibromo-3-chloropropane in water by the combination of hydrogen peroxide and UV radiation. Industrial & Engineering Chemistry Research 34(7), 2314-2323.

Hessler, D., Gorenflo, V. and Frimmel, F. (1993) Degradation of Aqueous Atrazine and Metazachlor Solutions by UV and UV/H_2_O_2_—Influence of pH and Herbicide Concentration Abbau von Atrazin und Metazachlor in wäßriger Lösung durch UV und UV/H_2_O_2_—Einfluß von pH und Herbizid‐Konzentration. Acta hydrochimica et hydrobiologica 21(4), 209-214.

Huie, R.E. and Clifton, C.L. (1990) Temperature dependence of the rate constants for reactions of the sulfate radical, SO_4_^-^, with anions. Journal of Physical Chemistry 94(23), 8561-8567.

Iwasaki, T., Masuda, Y., Ogihara, H. and Yamanaka, I. (2018) Direct Synthesis of Pure H_2_O_2_ Aqueous Solution by CoTPP/Ketjen-Black Electrocatalyst and the Fuel Cell Reactor. Electrocatalysis 9(2), 236-242.

Jasper, J.T. and Sedlak, D.L. (2013) Phototransformation of Wastewater-Derived Trace Organic Contaminants in Open-Water Unit Process Treatment Wetlands. Environmental Science & Technology 47(19), 10781-10790.

Jayson, G., Parsons, B. and Swallow, A.J. (1973) Some simple, highly reactive, inorganic chlorine derivatives in aqueous solution. Their formation using pulses of radiation and their role in the mechanism of the Fricke dosimeter. Journal of the Chemical Society, Faraday Transactions 1: Physical Chemistry in Condensed Phases 69, 1597-1607.

Jirkovský, J., Faure, V. and Boule, P. (1997) Photolysis of Diuron. Pesticide Science 50(1), 42-52.

King, J.F., Szczuka, A., Zhang, Z. and Mitch, W.A. (2020) Efficacy of ozone for removal of pesticides, metals and indicator virus from reverse osmosis concentrates generated during potable reuse of municipal wastewaters. Water research 176, 115744.

Ko, K.B., Lee, J.Y., Yoon, Y.H., Moon, T.H., Ahn, Y.H., Park, C.G., Min, K.S. and Park, J.H. (2009) Effects of nitrate on the UV photolysis of H_2_O_2_ for 2,4-dichlorophenol degradation in treated effluents. Desalination and Water Treatment 2(1-3), 6-11.

Lee, E., Shon, H.K. and Cho, J. (2014) Role of wetland organic matters as photosensitizer for degradation of micropollutants and metabolites. Journal of hazardous materials 276, 1-9.

Li, H., Wen, P., Itanze, D.S., Hood, Z.D., Adhikari, S., Lu, C., Ma, X., Dun, C., Jiang, L., Carroll, D.L., Qiu, Y. and Geyer, S.M. (2020) Scalable neutral H_2_O_2_ electrosynthesis by platinum diphosphide nanocrystals by regulating oxygen reduction reaction pathways. Nature Communications 11(1), 3928.

Li, W., Bonakdarpour, A., Gyenge, E. and Wilkinson, D.P. (2013) Drinking Water Purification by Electrosynthesis of Hydrogen Peroxide in a Power-Producing PEM Fuel Cell. ChemSusChem 6(11), 2137-2143.

Luo, H., Li, C., Wu, C. and Dong, X. (2015) In situ electrosynthesis of hydrogen peroxide with an improved gas diffusion cathode by rolling carbon black and PTFE. RSC Advances 5(80), 65227-65235.

Mandal, P.C., Bardhan, D.K., Sarkar, S. and Bhattacharyya, S.N. (1991) Oxidation of nickel (II) ethylenediaminetetraacetate by carbonate radical. Journal of the Chemical Society, Dalton Transactions (6), 1457-1461.

Marin, M.L., Lhiaubet-Vallet, V., Santos-Juanes, L., Soler, J., Gomis, J., Arques, A., Amat, A.M. and Miranda, M.A. (2011) A photophysical approach to investigate the photooxidation mechanism of pesticides: Hydroxyl radical versus electron transfer. Applied Catalysis B: Environmental 103(1), 48-53.

Mazellier, P., Leroy, E., De Laat, J. and Legube, B. (2003) Degradation of carbendazim by UV/H_2_O_2_ investigated by kinetic modelling. Environmental Chemistry Letters 1(1), 68-72.

Mazellier, P., Leroy, É. and Legube, B. (2002) Photochemical behavior of the fungicide carbendazim in dilute aqueous solution. Journal of Photochemistry and Photobiology A: Chemistry 153(1), 221-227.

Morgan, M.S., Van Trieste, P.F., Garlick, S.M., Mahon, M.J. and Smith, A.L. (1988) Ultraviolet molar absorptivities of aqueous hydrogen peroxide and hydroperoxyl ion. Analytica chimica acta 215, 325-329.

Nick, K., Schöler, H., Mark, G., Söylemez, T., Akhlaq, M., Schuchmann, H. and Von Sonntag, C. (1992) Degradation of some triazine herbicides by UV radiation such as used in the UV disinfection of drinking water. Aqua- Journal of Water Supply: Research and Technology 41(2), 82-87.

Olasehinde, E.F., Hasan, N., Omogbehin, S.A., Kondo, H. and Sakugawa, H. (2013) Hydroxyl radical mediated degradation of diuron in river water. The Journal of American Science 9(4), 29-34.

Oloman, C. and Watkinson, A. (1979) Hydrogen peroxide production in trickle-bed electrochemical reactors. Journal of Applied Electrochemistry 9(1), 117-123.

Peller, J.R., Mezyk, S.P. and Cooper, W.J. (2009) Bisphenol A reactions with hydroxyl radicals: diverse pathways determined between deionized water and tertiary treated wastewater solutions. Research on Chemical Intermediates 35(1), 21-34.

Pereira, V.J., Weinberg, H.S., Linden, K.G. and Singer, P.C. (2007) UV Degradation Kinetics and Modeling of Pharmaceutical Compounds in Laboratory Grade and Surface Water via Direct and Indirect Photolysis at 254 nm. Environmental Science & Technology 41(5), 1682-1688.

Sa, Y.J., Kim, J.H. and Joo, S.H. (2019) Active Edge-Site-Rich Carbon Nanocatalysts with Enhanced Electron Transfer for Efficient Electrochemical Hydrogen Peroxide Production. Angewandte Chemie International Edition 58(4), 1100-1105.

Sanches, S., Barreto Crespo, M.T. and Pereira, V.J. (2010) Drinking water treatment of priority pesticides using low pressure UV photolysis and advanced oxidation processes. Water research 44(6), 1809-1818.

Sehested, K., Rasmussen, O.L. and Fricke, H. (1968) Rate constants of OH with HO_2_, O_2_^-^, and H_2_O_2_^+^ from hydrogen peroxide formation in pulse-irradiated oxygenated water. The Journal of Physical Chemistry 72(2), 626-631.

Semitsoglou-Tsiapou, S., Templeton, M.R., Graham, N.J.D., Hernández Leal, L., Martijn, B.J., Royce, A. and Kruithof, J.C. (2016) Low pressure UV/H_2_O_2_ treatment for the degradation of the pesticides metaldehyde, clopyralid and mecoprop – Kinetics and reaction product formation. Water research 91, 285-294.

Shemer, H. and Linden, K.G. (2006) Degradation and by-product formation of diazinon in water during UV and UV/H_2_O_2_ treatment. Journal of hazardous materials 136(3), 553-559.

U.S. Energy Information Administration (2020) Table 5.6.A. Average Price of Electricity to Ultimate Customers by End-Use Sector. URL:<https://www.eia.gov/electricity/monthly/epm_table_grapher.php?t=epmt_5_6_a>.

Vogna, D., Marotta, R., Andreozzi, R., Napolitano, A. and d’Ischia, M. (2004) Kinetic and chemical assessment of the UV/H_2_O_2_ treatment of antiepileptic drug carbamazepine. Chemosphere 54(4), 497-505.

Wang, Y., Shi, R., Shang, L., Waterhouse, G.I.N., Zhao, J., Zhang, Q., Gu, L. and Zhang, T. (2020) High-Efficiency Oxygen Reduction to Hydrogen Peroxide Catalyzed by Nickel Single-Atom Catalysts with Tetradentate N_2_O_2_ Coordination in a Three-Phase Flow Cell. Angewandte Chemie International Edition 59(31), 13057-13062.

Weinstein, J. and Bielski, B.H. (1979) Kinetics of the interaction of perhydroxyl and superoxide radicals with hydrogen peroxide. The Haber-Weiss reaction. Journal of the American Chemical Society 101(1), 58-62.

Wong, C.C. and Chu, W. (2003) The direct photolysis and photocatalytic degradation of alachlor at different TiO_2_ and UV sources. Chemosphere 50(8), 981-987.

Wu, C., Shemer, H. and Linden, K.G. (2007) Photodegradation of Metolachlor Applying UV and UV/H_2_O_2_. Journal of Agricultural and Food Chemistry 55(10), 4059-4065.

Wu, Z., Yang, L., Tang, Y., Qiang, Z. and Li, M. (2021) Dimethoate degradation by VUV/UV process: Kinetics, mechanism and economic feasibility. Chemosphere 273, 129724.

Xia, C., Xia, Y., Zhu, P., Fan, L. and Wang, H. (2019) Direct electrosynthesis of pure aqueous H_2_O_2_ solutions up to 20% by weight using a solid electrolyte. Science 366(6462), 226-231.

Yamanaka, I., Hashimoto, T., Ichihashi, R. and Otsuka, K. (2008) Direct synthesis of H_2_O_2_ acid solutions on carbon cathode prepared from activated carbon and vapor-growing-carbon-fiber by a H_2_/O_2_ fuel cell. Electrochimica Acta 53(14), 4824-4832.

Yamanaka, I. and Murayama, T. (2008) Neutral H_2_O_2_ Synthesis by Electrolysis of Water and O_2_. Angewandte Chemie International Edition 47(10), 1900-1902.

Yamanaka, I., Onizawa, T., Suzuki, H., Hanaizumi, N. and Otsuka, K. (2006) Electrocatalysis of heat-treated Mn–porphyrin/carbon cathode for synthesis of H_2_O_2_ acid solutions by H_2_/O_2_ fuel cell method. Chemistry Letters 35(12), 1330-1331.
